# Co-Occurrence of DON and Emerging Mycotoxins in Worldwide Finished Pig Feed and Their Combined Toxicity in Intestinal Cells

**DOI:** 10.3390/toxins11120727

**Published:** 2019-12-11

**Authors:** Abdullah Khan Khoshal, Barbara Novak, Pascal G. P. Martin, Timothy Jenkins, Manon Neves, Gerd Schatzmayr, Isabelle P. Oswald, Philippe Pinton

**Affiliations:** 1 Toxalim (Research Center in Food Toxicology), Université de Toulouse, INRAE, ENVT, INP-Purpan, UPS, 180 chemin de tournefeuille, Cedex 3, F-31027 Toulouse, France; abdullah.khoshal@inra.fr (A.K.K.); pascal.martin@inra.fr (P.G.P.M.); manon.neves@inra.fr (M.N.); 2 BIOMIN Research Center, Technopark 1, 3430 Tulln, Austria; barbara.novak@biomin.net (B.N.); timothy.jenkins@biomin.net (T.J.); gerd.schatzmayr@biomin.net (G.S.)

**Keywords:** global survey, finished pig feed, co-occurrence, emerging mycotoxins, DON, toxicity, combined toxicity, IPEC-1

## Abstract

Food and feed can be naturally contaminated by several mycotoxins, and concern about the hazard of exposure to mycotoxin mixtures is increasing. In this study, more than 800 metabolites were analyzed in 524 finished pig feed samples collected worldwide. Eighty-eight percent of the samples were co-contaminated with deoxynivalenol (DON) and other regulated/emerging mycotoxins. The Top 60 emerging/regulated mycotoxins co-occurring with DON in pig feed shows that 48%, 13%, 8% and 12% are produced by *Fusarium*, *Aspergillus*, *Penicillium* and *Alternaria* species, respectively. Then, the individual and combined toxicity of DON and the 10 most prevalent emerging mycotoxins (brevianamide F, cyclo-(L-Pro-L-Tyr), tryptophol, enniatins A1, B, B1, emodin, aurofusarin, beauvericin and apicidin) was measured at three ratios corresponding to pig feed contamination. Toxicity was assessed by measuring the viability of intestinal porcine epithelial cells, IPEC-1, at 48-h. BRV-F, Cyclo and TRPT did not alter cell viability. The other metabolites were ranked in the following order of toxicity: apicidin > enniatin A1 > DON > beauvericin > enniatin B > enniatin B1 > emodin > aurofusarin. In most of the mixtures, combined toxicity was similar to the toxicity of DON alone. In terms of pig health, these results demonstrate that the co-occurrence of emerging mycotoxins that we tested with DON does not exacerbate toxicity.

## 1. Introduction

Mycotoxins are low molecular weight fungal secondary metabolites that trigger a detrimental response when ingested by humans and animals. They are mainly produced by filamentous fungi belonging to *Aspergillus*, *Fusarium* and *Penicillium* species [1]. Mycotoxin contamination can occur all along the food chain from field to storage, including the food process. This depends upon the requirements of fungi, and *Fusarium* mostly occurs in the field, whereas *Aspergillus* and *Penicillium* mostly occurs during storage.

Because of their toxicity and occurrence, deoxynivalenol (DON), zearalenone, aflatoxins, ochratoxin A, patulin, fumonisins and T-2/HT-2 toxins are regulated in Europe. For example, the maximum recommendations limits that are set up for complete piglet feed are 0.9, 0.1, 0.05, 5 and 0.25 mg/kg feed for DON, zearalenone, ochratoxin, fumonisins and T2 + HT2, respectively [2,3]. However, in addition to regulated mycotoxins, many other fungal secondary metabolites are being identified in food and feed [4,5]. Metabolites that are neither routinely determined, nor legislatively regulated, have been defined as ‘emerging mycotoxins’ [6], while the derivatives of regulated mycotoxins that are undetectable using conventional analytical techniques due to their modified structure, are defined as ‘modified/masked mycotoxins’ [4,7]. Recent findings showed that more than 70% of the world’s cereal grains are contaminated by mycotoxins [8,9], often in a mixture [10].

Among regulated mycotoxins, DON very frequently contaminates cereals (wheat, barley, oats, rye and maize, and less frequently rice, sorghum and triticale) and cereal-based food and feed. DON belongs to the group of B-trichothecenes, and is one of the most widely distributed contaminants in human food and animal feed. In a total of more than 25,000 samples collected from 28 European countries between 2007 and 2014, DON was found in 47% of 4000 feed samples and 45% of 1621 unprocessed grains with no defined end use, respectively [11]. Even though DON is considered as a non-carcinogenic compound [12], the maximum level of this toxin in food and feed have been set up in different countries. For example, in complete piglet feed, the maximum limits are 0.9, 1 and 5 mg/kg feed in Europe, Canada and the USA, respectively [2,13]. Exposure to high concentrations of DON is associated with diarrhea, vomiting (emesis), leukocytosis and gastrointestinal bleeding. Chronic exposure affects growth, immunity and intestinal barrier function in animals [14,15,16]. This toxin interacts with the peptidyl transferase region of the 60S ribosomal subunit, inducing ‘ribotoxic stress’, resulting in the activation of mitogen-activated protein kinases (MAPKs) and their downstream pathways [14,17].

Among emerging mycotoxins, those that occur most frequently are enniatins (ENNs), beauvericin (BEA), apicidin (API), aurofusarin (AFN), culmorin, butenolide, fusaric acid, moniliformin, fusaproliferin and emodin (EMO). They are produced by *Fusarium* species except EMO, which is produced by *Aspergillus* species [6,18]. ENNs and BEA were detected in food (63% and 80%), feed (32% and 79%), and unprocessed grains (24% and 46%) collected between 2010 and 2014 in 12 European countries [19]. AFN, API, brevianamide-F (BRV-F), EMO and tryptophol (TRPT) were also found in pig feed (80%, 52%, 65%, 63% and 75%) [20], Egyptian animal feed (73%, 17%, 86%, 98% and 90%) [21] and feed raw materials (84%, 55%, 5%, 74%, 59%) [22].

Multiple mycotoxins are frequently present in food and feed [10]. The co-occurrence of DON, aflatoxins, fumonisins, zearalenone and other fungal secondary metabolites in maize seeds and grains, as well as in animal feed, has been reported [21,22,23]. The presence of different fungi on the same raw material, the ability of fungal species to produce several toxins, as well as the various commodities present in completed feed, can explain this multiple contamination [24,25]. Compound feed is particularly prone to multiple contaminations, as it typically contains a mixture of several raw materials.

The co-occurrence of mycotoxins is challenging for at least two reasons: (i) The toxicity of mycotoxins when present together cannot always be predicted based upon their individual toxicity and (ii) the risk assessment is performed on a chemical-by-chemical basis [24,25]. Scientific interest in the toxicity of these mixtures of mycotoxins is currently increasing rapidly [26,27,28,29]. Several studies have investigated the combined toxicity of regulated mycotoxins on the intestine [30,31,32,33], but the combined effect of regulated and other mycotoxins is poorly documented [34,35].

Among farm animals, pig is one of the most sensitive species to mycotoxins [36]. As feed raw materials are potentially contaminated by several fungi at a time, and completed feed is made from various commodities, pig can be exposed, through its rich cereal diet, to high concentrations of mixtures of mycotoxins [10,37]. The sanitary and economic losses due to mycotoxin contamination are important in the pig industry, even if they are hard to estimate precisely [38].

The aims of this study were thus to (i) determine the prevalence and concentration of mycotoxins present in finished pig feed and (ii) assess the intestinal combined toxicity of DON in mixture with the 10 most prevalent emerging mycotoxins present in pig feed using realistic ratios.

## 2. Results

### 2.1. Occcurrence and Abundance of Emerging Mycotoxins and DON in Finished Pig Feed

**Figure 1:**
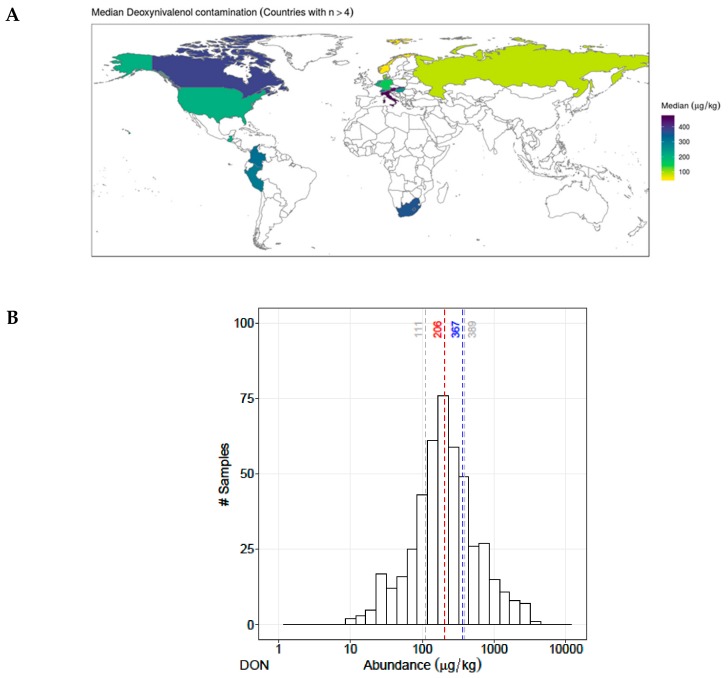
(**A**) Worldwide contamination of deoxynivalenol (DON). Concentration of DON is highlighted by colors, where yellow indicates > 100 µg/kg, green > 200 µg/kg, dark green > 300 and dark blue indicates > 400 µg/kg. (**B**) Abundance of DON in pig finished feed (P25, median, mean, P75). X axis represents the distribution of the concentration, Y axis describes the number of contaminated samples.

A total of 524 finished pig feed samples collected worldwide were analyzed, and more than 235 different metabolites were detected, including regulated mycotoxins, emerging mycotoxins and modified/masked mycotoxins. Table 1 lists the 60 most prevalent fungal metabolites that contaminated more than 20% of the finished pig feed samples. Among regulated mycotoxins, DON was detected in 463 samples (88%), mostly in the Northern Hemisphere and in relatively similar concentrations in samples from all countries (median concentration 206 µg/kg) (Figure 1A,B).

All DON-contaminated samples were co-contaminated by other mycotoxins. The distribution of the samples was checked. Table 1 gives the descriptive statistics for DON and the most abundant metabolites that co-occur with DON.

From this list, the most prevalent emerging mycotoxins co-occurring with DON and which were commercially available were selected for the toxicological studies. Because of its high toxicity [39], apicidin (API) was included in the list. The worldwide distributions of these compounds are presented in Supplementary Figure S1. Except for API, which was detected in only 67% of DON-contaminated samples, the selected emerging mycotoxins were present in more than 87% of them (Table 1). Three compounds API, emodin (EMO) and beauvericin (BEA) were detected in a median concentration range of 5 to 10 µg/kg feed. The median concentration of four metabolites, enniatins A1, B, B1 (ENN-A1, B, B1) and brevianamide F (BRV-F), was in the range of 15-40 µg/kg feed, and the last three compounds aurofusarin (AFN), cyclo-(L-Pro-L-Tyr) (Cyclo) and tryptophol (TRPT) had median concentrations close to 200 µg/kg feed, like DON (Supplementary Figure S2). Despite their high co-occurrence with DON, with the exception of AFN, and to a lesser extent BEA, the concentration of these mycotoxins showed limited correlation with DON concentration (Table 1 and Supplementary Figure S3).

### 2.2. Intestinal Toxicity of Emerging Mycotoxins Found in Pig Feed, alone or Combined with DON

#### 2.2.1. Individual Toxicity of DON and Emerging Mycotoxins

**Figure 2:**
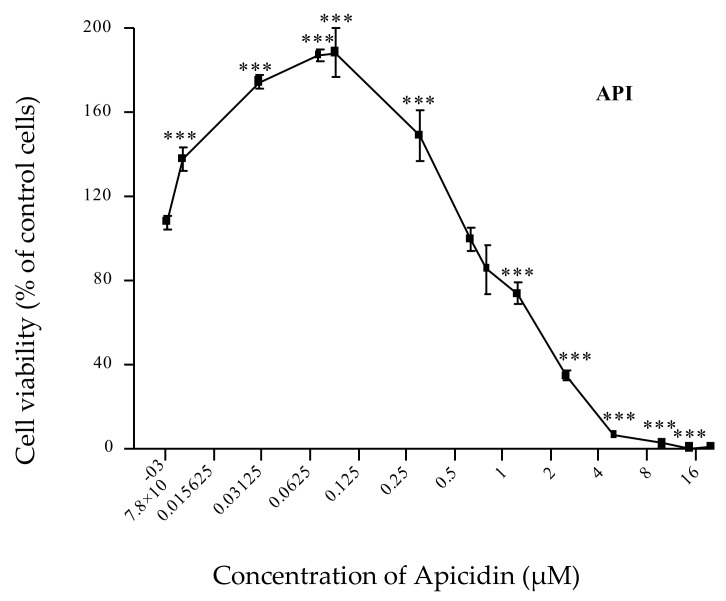
Dose effect curve of individual toxicity of apicidin (API). Data are mean ± SEM of three biological replicates. Bonferroni multiple comparison test. Significant difference between control and different doses of API *** *p* < 0.001.

The individual intestinal toxicity of 10 selected emerging mycotoxins, as well as that of DON, was first analyzed at a wide range of concentrations (Supplementary Figure S4). As shown in Supplementary Figure S4, all tested metabolites exhibited dose-dependent toxicity toward intestinal epithelial cells, except BRV-F, Cyclo and TRPT, that were not toxic for this porcine cell line. AFN, EMO and ENN-B1 reduced the viability of IPEC-1, but their toxicity was less than that of DON. The toxicity of BEA and ENN-B was close to that of DON, whereas API and ENN-A1 were more toxic than DON. As shown in Figure 2, low doses of API (0.01–0.3 µM) significantly stimulated the proliferation of IPEC-1. When the doses leading to a 50% reduction in the cell viability (IC_50_) of these emerging mycotoxins were compared with the dose of DON, then BRV-F, Cyclo and TRPT were classified as non-toxic metabolites, while EMO, AFN and ENN-B1 were given as moderately toxic metabolites, and finally API, ENN-A1, ENN-B and BEA as highly toxic metabolites (Table 2).

#### 2.2.2. Combined Toxicity of DON and Emerging Mycotoxins

Next, the combined toxicity of DON and the selected emerging mycotoxins was assessed. As mentioned above, the concentration of these secondary metabolites was not correlated with the concentration of DON (Table 1 and Supplementary Figure S3). Thus, to account for the different situations to which pigs can be exposed, three ratios were tested (Supplementary Table S1). Ratio 1 was calculated using the P25 (1st quartile) concentration of the emerging mycotoxin and P75 (3rd quartile) concentration of DON. Ratio 2 was calculated using the median (2nd quartile) concentration of DON and each emerging mycotoxin. Ratio 3 was calculated using the P75 concentration of the emerging mycotoxin and P25 concentration of DON. For each ratio, serial dilutions were tested to obtain a dose-effect curve that encompassed the realistic concentrations of the mixture of DON and the tested metabolites. 

#### 2.2.3. Combined Toxicity of DON and the Non-Toxic Secondary Metabolites (BRV-F, Cyclo and TRPT)

**Figure 3:**
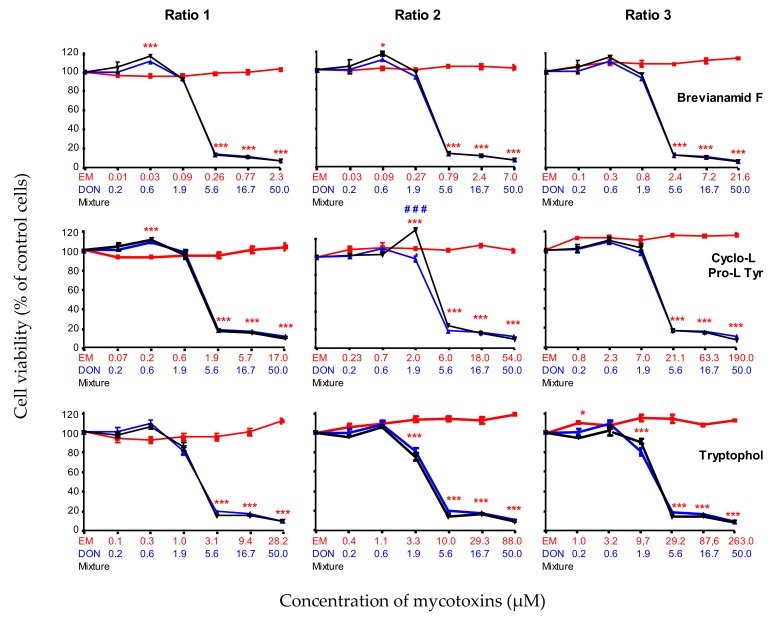
Dose-effect curves of deoxynivalenol (DON) (blue lines and symbols), emerging mycotoxins (brevianamide-F (BRV-F), cyclo-(L-Pro-L-Tyr) (Cyclo) and tryptophol (TRPT)) alone (red lines and symbols), or in combination with DON (black lines and symbols) at different ratios: ratio 1 was calculated from the P25 concentration of emerging mycotoxin and P75 concentration of DON; ratio 2 was calculated from the median concentration of emerging mycotoxin and DON; ratio 3 was calculated from the P75 concentration of emerging mycotoxin and the P25 concentration of DON. Six serial dilutions of each ratio were tested (Emerging mycotoxin alone, DON alone, mixture). Data are mean ± SEM of three biological replicates. Bonferroni multiple comparison test. Significant difference between emerging mycotoxins alone and the mixtures *** *p* < 0.001. Significant difference between DON alone and the mixtures # # # *p* < 0.001.

First, the combined toxicity of DON and the ‘non-toxic’ secondary metabolites BRV-B, Cyclo and TRPT were analyzed. As shown in Figure 3, whatever the ratios tested, the toxicity of the combination of DON and the compound being tested was similar to the toxicity of DON alone.

#### 2.2.4. Combined Toxicity of DON and the Moderately Toxic Secondary Metabolites (AFN, EMO and ENN-B1)

**Figure 4:**
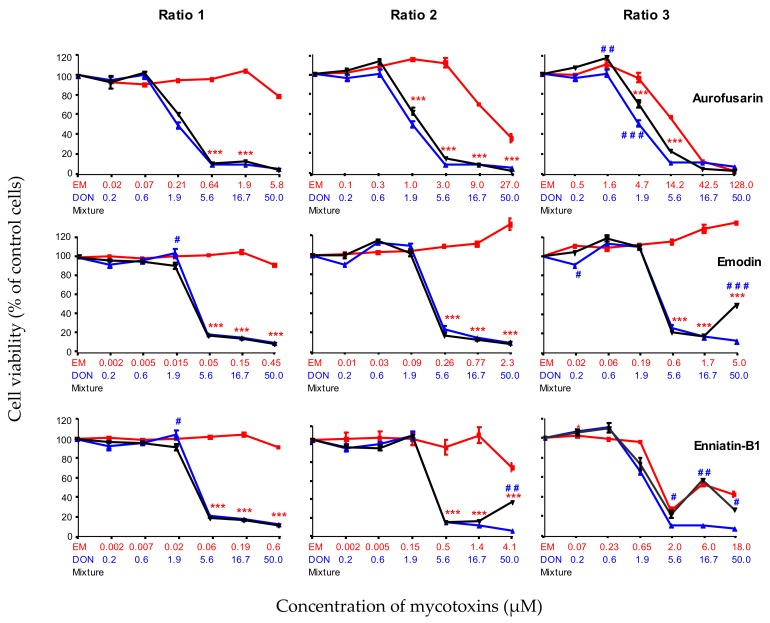
Dose-effect curves of DON (blue lines and symbols), emerging mycotoxins (AFN, EMO and ENN-B1) alone (red lines and symbols) or in combination with DON (black lines and symbols) at different ratios: ratio 1 was calculated from the P25 concentration of emerging mycotoxin and P75 concentration of DON; ratio 2 was calculated from the median concentration of emerging mycotoxin and DON; ratio 3 was calculated from the P75 concentration of emerging mycotoxin and P25 concentration of DON. Six serial dilutions of each ratio were tested (Emerging mycotoxin alone, DON alone, mixture). Data are mean ± SEM of three biological replicates. Bonferroni multiple comparison test. Significant difference between emerging mycotoxins alone and the mixtures *** *p* < 0.001. Significant difference between DON alone and the mixtures # # # *p* < 0.001.

Next, the combined toxicity of DON and the moderately toxic metabolites AFN, EMO and ENN-B1 was assessed (Figure 4). The toxicity of AFN, EMO and ENN-B1 was minimal when used at ratio 1. In these conditions, the toxicity of the combination of DON and emerging toxins was similar to the toxicity of DON alone. Ratio 2 reached toxic concentrations of AFN and ENN-B1, but the toxicity of the mixture was similar to the toxicity of DON alone, except at the highest concentration of ENN-B1 (4.1 µM), where the toxicity of the mixture (ENN-B1 4.1 µM + DON 50 µM) was higher than the toxicity of ENN-B1 alone, but lower than the toxicity of DON alone. 

When cytotoxicity was tested at ratio 3, the toxicity of AFN + DON was identical to the one of DON alone except at the concentrations DON 0.6 µM + AFN 1.6 µM and DON 1.9 µM + AFN 4.7 µM, when the toxicity of the mixture was slightly lower than the toxicity of DON alone. At this ratio, EMO alone was still not toxic, and even induced proliferation (up to 130% of treated cells). 

The combined toxicity of DON and EMO was similar to the toxicity of DON alone except at the highest concentration of EMO, when the mixture of DON (50 µM) and EMO (5 µM) was still less toxic than DON alone. The combined toxicity of ENN-B1 + DON was the same as the toxicity of DON alone, except at the highest concentrations of ENN-B1 (6 and 18 µM), when the toxicity of the mixture was the same as the toxicity of ENN-B1 alone, but lower than the toxicity of DON alone.

In conclusion, our data showed that the toxicity of the combination of DON and emerging mycotoxins such as AFN, EMO and ENN-B1 was similar to or lower than the toxicity of DON alone, whatever the ratio used.

#### 2.2.5. Combined Toxicity of DON and The Highly Toxic Secondary Metabolites (ENN-B, BEA, ENN-A1and API)

**Figure 5:**
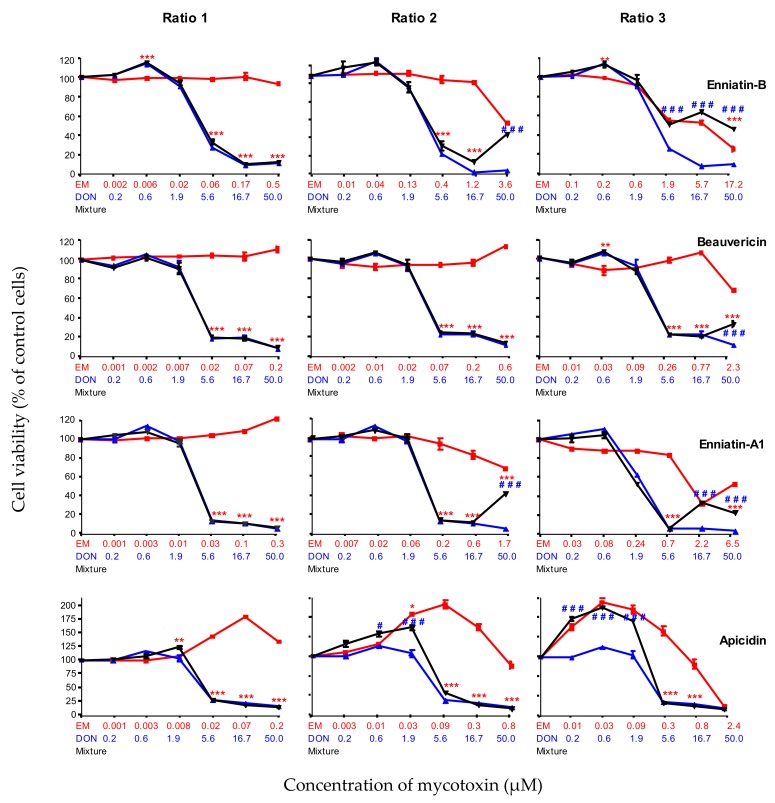
Dose-effect curves of DON (blue lines and symbols), emerging mycotoxins (ENN-B, BEA, ENN-A1 and API) alone (red lines and symbols) or in combination with DON (black lines and symbols) at different ratios: ratio 1 was calculated from the P25 concentration of the emerging mycotoxin and the P75 concentration of DON; ratio 2 was calculated from the median concentration of emerging mycotoxin and DON; ratio 3 was calculated from the P75 concentration of emerging mycotoxin and the P25 concentration of DON. Six serial dilutions of each ratio were tested (Emerging mycotoxin alone, DON alone, mixture). Data are mean ± SEM of three biological replicates. Bonferroni multiple comparison test. Significant difference between emerging mycotoxins alone and the mixtures *** *p* < 0.001. Significant difference between DON alone and the mixtures # # # *p* < 0.001.

The combined toxicity of DON and highly toxic compounds (ENN-B, BEA, ENN-A1 and API) was also analyzed at different ratios (Figure 5). At the first tested ratio (ratio 1), ENN-B, BEA, ENN-A1 and API were not toxic, and their combined toxicity in the presence of DON was similar to the toxicity of DON alone. Ratio 2 reached toxic concentrations of ENN-B, ENN-A1 and API. 

For ENN-B and ENN-A1, the toxicity of the mixture was similar to the toxicity of DON alone, except at the highest concentrations of ENN-B (3.6 µM) and ENN-A1 (1.7 µM). The combined toxicity of DON (50 µM) + ENN-B (3.6 µM) was similar to the toxicity of ENN-B alone, but lower than the toxicity of DON alone, whereas the combined toxicity of DON (50 µM) + ENN-A1 (1.7 µM) was higher than the toxicity of ENN-A1 alone, but still lower than the toxicity of DON alone. The combined toxicity of API and DON was similar to the toxicity of DON only for the higher doses. For the doses lower than DON (5.6 µM) + API (0.09 µM) the combined toxicity was lower than the toxicity of DON alone, but higher than API alone. 

At ratio 3, the combined toxicity of DON and ENN-B displayed a different characteristic. At a high concentration, the combined toxicity of DON (50 µM) + ENN-B (17.2 µM) was lower than the toxicity of either DON alone or ENN-B alone. At this ratio, the toxicity of the mixture of DON and BEA was similar to the toxicity of DON alone, except at the highest concentration of BEA (2.3 µM) when mixed with DON (50 µM), where the toxicity was higher than that of BEA alone, but lower than that of DON alone. The combined toxicity of DON (16.7µM) + ENN-A1 (2.2 µM) at ratio 3 was similar to the toxicity of ENN-A1 alone, but lower than the toxicity of DON alone; the toxicity of DON (50 µM) + ENN-A1 (6.5 µM) was higher than the toxicity of ENN-A1 alone, but lower than the toxicity of DON alone. Finally, the combined toxicity of DON and API, at concentrations lower than DON (5.6 µM) + API (0.3 µM), was always similar to API alone, but lower than DON alone, whereas at higher concentrations, it was similar to the one of DON alone and higher than the one of API alone. As mentioned above, a proliferation of IPEC-1 cells was observed at low concentrations of API.

In conclusion, our data showed that the toxicity of the combination of DON and highly toxic emerging mycotoxins such as ENN-B, BEA, ENN-A1 and API, whatever the ratio used, was similar to or lower than the toxicity of DON alone.

## 3. Discussion

Progress in analytical methods enabled the discovery of numerous fungal secondary metabolites that are the subject of increasing attention today due to their prevalence in human food and animal feed [19,20]. In the present study, 524 samples of finished pig feed were analyzed. In addition to regulated mycotoxins such as DON, zearalenone and fumonisin B1, less known secondary metabolites were detected. 

As already described in other surveys [20,21,22], BRV-F, Cyclo, TRPT, ENNs, EMO, BEA and AFN, culmorin, and moniliformin were highly prevalent emerging mycotoxins detected in more than 85% of pig feed samples. The diversity of the metabolites detected is very likely related to the wide range of fungal species that contaminate the raw materials used to make pig feed. Indeed, *Fusarium* species produce ENNs (A1, B and B1), BEA, AFN, API, culmorin and moniliformin, while *Penicillium* species produce BRV-F and Cyclo, *Aspergillus* EMO and *Acremonium* TRPT [22,40].

The toxicity of these new poorly documented metabolites was also investigated in the present study. The results of our analyses showed that, even at high concentrations of up to 300 µM, BRV, Cyclo and TRPT are not toxic to intestinal cells. Similar results were recently obtained using another porcine intestinal cell line, IPEC-J2, and a different readout, cellular protein content [20]. Interestingly, at a much higher concentration (2 mM), TRPT induced DNA damage in HepG2, A549 and THP-1 cells [41]. AFN and EMO were identified as moderately toxic compounds at relative IC_50_ values of 19.1 µM and 19 µM, respectively. These emerging mycotoxins were found to be more toxic for IPEC-J2 with relative IC_50_ of 9.3 µM and 13.1 µM, respectively [20]. On the other hand, human multiple myeloma blood cells were less sensitive to EMO (IC_50_ 38 µM) [42]. According to their IC_50_, ENNs were ranked in the following order of toxic potency ENN-A1 > ENN-B > ENN-B1. Similar ranking was reported for HT-29 [43] and IPEC-J2 [44]. ENN-A1 is also more toxic than ENN-B1 for Caco-2 and HepG2, but ENN-B displayed no toxicity at all [43]. The mechanism of toxicity of ENNs is related to their ionophoric properties [19] that facilitate the transport of mono- or divalent cations such as K^+^ or Ca^2+^ across membranes, but the relative sensitivity of the different cell lines to the different ENNs is still not understood. The high toxicity of BEA has already been reported in other cell lines of human and porcine origin, including Caco-2, HT-29 and IPEC-J2 [20,43]. BEA also has ionophoric properties and increase ions permeability (Na^+^ K^+^ and Ca^2+^) in biological membrane, this mechanism of action participates to the toxicity of this mycotoxin [6,19,44].

Data on the toxicity of API are very limited. We confirmed the toxicity of this metabolite when used at high concentration (> 0.5 µM) [20,39]; we also observed that low concentrations of API (<0.1 µM) stimulate the proliferation of porcine intestinal cells. The proliferative effect of low doses of some mycotoxins has already been described. For example, up to 200% proliferation has been observed in lymphocytes and splenocytes exposed to low doses (> 0.1µM) of DON, nivalenol, aflatoxin B1 or fumonisin B1 [45]. Because of its high prevalence and its low IC_50_, it would be of great interest to deepen our knowledge of API. The toxicity of other very prevalent metabolites, such as culmorin and moniliformin, could not be evaluated in this study because they were not commercially available in the quantities required for cellular experiments.

Different studies have described the co-occurrence of mycotoxins in cereals and other raw materials used for animal feed in several regions of the globe [21,46,47]. In the present study, we investigated the co-occurrence of mycotoxins in pig feed. We identified 60 metabolites that co-occur with DON in more than 20% of samples, confirming the prevalence of co-contamination, and as a result, the exposure of animals to a mixture of mycotoxins. Our results are in accordance with those of previous surveys of raw materials and finished feed [20,46]. Although the exact composition of the analyzed pig feed is not known, the main component of the majority of the samples is maize. The extraction efficiencies are between 85%-95% determined in seven different matrices [48]. However, the focus of our study is to provide a general picture of the worldwide contamination of pig feed and to test realistic ratios of fungal compounds in vitro. 

Despite the very high frequency of co-contamination by DON and emerging mycotoxins, the correlation between the concentrations of DON and emerging mycotoxins was very low. The correlation between DON and emerging mycotoxins is poorly documented. In winter wheat, Blandino and co-workers [49] addressed the correlation between DON and other mycotoxins produced by *Fusarium graminearum* and *F. culmorum*, and showed that correlations between DON and either culmorin or moniliformin were significant (0.94 and 0.42, respectively). By contrast, the correlations between DON and AFN or BEA were not significant (0.2 and -0.14, respectively). Correlations between the concentration of DON and its modified forms enabled EFSA to calculate ratios between these different toxins [11]. 

In the present study, the absence of correlation between the concentrations of the emerging mycotoxins and DON could be explained by the diversity of fungi that produce the various metabolites via different biosynthetic pathways. Furthermore, pigs feed is made of different raw materials, which also explains the lack of correlation between the amounts of the different fungal metabolites involved.

The main objective of the present study was to assess the combined toxicity of DON and emerging mycotoxins. As no correlation was found, to encompass the situations to which animals may be exposed, different plausible ratios were tested. These ratios were based on the P25, the median and the P75 concentrations of DON and emerging mycotoxins observed in pig feed, because these summary statistics are robust to extreme outlier values. In most cases, we observed that when the non-toxic metabolites (BRV, Cyclo and TRPT) were present in mixture with DON, whatever the doses or the ratio, the effect was driven by DON. We observed a similar trend for the combined toxicity of DON with moderate and highly toxic metabolites ENNs, BEA, API, AFN and EMO. The effect of the mixtures was mostly similar to the effect of DON alone. The only exception was when very high concentrations of DON were used, in which cases surprisingly, the toxicity of the mixture was lower than the toxicity of DON alone.

To the best of our knowledge, this is the first study to investigate the interactions of different mycotoxins using plausible ratios. Most published studies used toxins of equal toxicity, regardless of their concentration in food or feed. For example, exposure of Caco-2 or IPEC-1 cells to low doses of DON, combined with isotoxic concentrations of nivalenol and/or their acetylated derivatives, led to a synergistic effect [31,32]. Similarly, mixtures of ENNs as well as mixtures of DON, ENNs and alternariol managed to induce synergistic cytotoxicity in Caco-2 cells [50,51]. A synergistic inflammatory effect was also observed in porcine intestinal explants co-exposed to DON and nivalenol [33].

In the future, these original data on the intestinal toxicity of realistic mixtures of DON and emerging mycotoxins should be completed by toxicity studies on other types of cells to account for all target organs. In vivo experiments are also needed to confirm the in vitro data. More widely, the effects of other mixtures of mycotoxins or of mycotoxins with other food contaminants should be investigated at realistic doses. As the pig is a good model for human toxicity studies of food contaminants [52], the results would be useful to estimate the effects of similar mixtures of toxins on human health. Better knowledge of the occurrence and toxicity of the real mixtures present in food is a precondition for the assessment of health risk [29].

## 4. Conclusions

This global survey of finished pig feed confirmed that such feed is co-contaminated by DON and emerging mycotoxins. However, despite the high percentages of co-occurrence, no correlation was found between the concentration of DON and most of these emerging mycotoxins. Using ratios based on the concentration of DON and emerging mycotoxins in feed, we observed that the toxicity of most of the mixtures was similar to the toxicity of DON alone. This demonstrates that, when these emerging mycotoxins are present together with DON, the toxicity of the mixture is not exacerbated.

## 5. Materials and Methods

### 5.1. Extraction and Analysis of Metabolites

A total of 524 finished pig feed samples were collected from 2014 to 2018 on the world market, but most in Europe (76.5%) and North America (15.8%) and fewer in Asia (3.2%), South Africa (1.5%), Australia (2 samples) and some of unknown origin (2.5%) (Figure 1 and Supplementary Figure S1), and more than 800 analytes, including fungal and bacterial secondary metabolites, were sought. Samples were provided by the BIOMIN Mycotoxin Survey, and the analyses were performed using the Liquid chromatography–mass spectrometry/mass spectrometry (LC–MS/MS) multi-mycotoxin method described by Malachova [5]. 

A QTrap5500 LC-MS/MS System (Applied Biosystems, Foster City, CA, USA) equipped with a TurboIonSpray electrospray ionization (ESI) source and a 1290 Series ultra high performance liquid chromatography (UHPLC) System (Agilent Technologies, Waldbronn, Germany) was used for detection and quantification of the analytes (Supplementary Figures S5 and S6). Chromatographic separation was performed on a Gemini^®^ C18-column (150 × 4.6 mm i.d., 5 μm particle size) equipped with a C18 security guard cartridge (4 × 3 mm i.d.) (all from Phenomenex, Torrance, CA, USA) at 25 °C. Elution was carried out in binary gradient mode. Both mobile phases contained 5 mm ammonium acetate (NH_4_CH_3_CO_2_) and were composed of methanol/water/acetic acid in a ratio of 10:89:1 (*v*/*v*/*v*; eluent A) or 97:2:1 (*v*/*v*/*v*; eluent B). After an initial time of 2 min at 100% A, the proportion of B was increased linearly to 50% within 3 min. Further linear increase of B to 100% within 9 min was followed by a hold-time of 4 min at 100% B and 2.5 min column re-equilibration at 100% A. The flow rate was 1000 μL/min. ESI-MS/MS was performed in the scheduled multiple reaction monitoring (sMRM) mode both in positive and negative polarities in two separate chromatographic runs. The sMRM detection window of each analyte was set to the respective retention time ± 27 s and ± 42 s in positive and in negative mode, respectively. The target scan time was set to 1 s. Confirmation of positive analyte identification is obtained by the acquisition of two sMRMs per analyte (with the exception of moniliformin and 3-nitropropionic acid, that each exhibit only one fragment ion), which yields 4.0 identification points according to commission decision 2002/657/EC (EU, 2002).

A total of 235 mycotoxins and other fungal secondary metabolites were detected and quantified in the samples of finished pig feed analyzed. The threshold of relevant concentrations was set at > 1.0 μg/kg or the limit of detection, whichever was higher. Samples were collected only by trained staff, or after the instruction of untrained staff according to a protocol. A minimum of 500 g homogenized sample was sent to the laboratory of the Institute of Bioanalytics and Agro-Metabolomics at the University of Natural Resources and Life Sciences Vienna (BOKU) in Tulln, Austria. Samples were milled and extracted with a mixture of acetonitrile, water and acetic acid (79:20:1, per volume) on a shaker for 90 min. The solution was centrifuged, after which the supernatant was diluted and injected into an LC-MS/MS system (electrospray ionization and mass spectrometric detection using a quadrupole mass filter). Quantification was done by comparing an external calibration using a multi-analyte stock solution. During the period 2014 to 2018, the number of substances measured using this method increased each year, and more substances were included in the survey. Nevertheless, the list of compounds investigated in this manuscript were those measured in 2014. All concentration data were collected in a single file, and sample information such as sampling year and month, country and region of origin and sample matrix were added for subsetting. Data were imported and analyzed in R v 3.5.1 mainly using tidy-verse packages (www.tidyverse.org). Spearman correlation coefficients and associated *p*-values were calculated with the corr.test function from the psych R package. Data were plotted (including maps) using ggplot2.

### 5.2. Toxins

For the cytotoxicity test, toxins were purchased from Sigma (St Quentin Fallavier, France): deoxynivalenol (DON) (purity > 98%), tryptophol (TRPT) (purity > 97%), apicidin (API) (purity > 98%) and emodin (EMO) (purity > 90%). Enniatins (ENNs) (A1, B, B1, purity > 99%), cyclo-(L-Pro-L-Tyr) (Cyclo) (purity > 98%), and brevianamide-F (BRV-F) (purity > 95%) were purchased from BioAustralis (Smithfield, Australia), beauvericin (BEA) (purity > 95%) and aurofusarin (AFN), (purity > 97%) were purchased from Santa Cruz Biotechnology (Dallas, TX, USA). All mycotoxins were dissolved in dimethyl sulfoxide (DMSO) (Sigma) to prepare stock solutions stored at −20 °C. Working dilutions were freshly prepared in cell culture medium for each experiment.

To convert the concentration in the pig feed into the concentration to which intestinal cells might be exposed, we assumed, as already done in previous studies [53], that mycotoxins were ingested in one meal, diluted in 1 L of gastrointestinal fluid and were entirely bioaccessible. Next, the ratio of DON to emerging mycotoxins was calculated based on three plausible scenarios according to the concentration of DON and emerging mycotoxins in the feed (Supplementary Table S1). 

Several 3-fold dilutions of each individual toxin and mixtures at different ratios were performed to account for the concentrations present in feed.

### 5.3. Cell Culture and Cytotxicity Assay

IPEC-1, derived from the small intestine of a newborn unsuckled piglet were maintained in complete medium (Dulbecco’s modified Eagle’s medium (DMEM)/Ham’s F12 medium (Sigma) plus 1% Penicillin Streptomycin (Eurobio, Courtaboeuf, France), 5% fetal bovine serum (FBS) (Eurobio), 1% L-glutamine (Eurobio), 5 μg/L epidermal growth factor (EGF) (Becton–Dickinson, Le Pont de Claix, France), and 1% ITS solution (insulin (5 μg/mL), transferrin (5 μg/mL), selenium (5 ng/mL), (Sigma Aldrich)) at 39 °C under 5% CO_2_, as previously described [54].

For the cytotoxicity experiments, cells were seeded in 96-white-well flat-bottom cell culture plates (Costar, Cambridge, MA, USA) at a rate of 10^4^ cells per well in 100 µL culture medium. After 24 h, the medium was replaced by complete medium without FBS containing the mycotoxins and incubated for a further 48 h. Toxicity was then assessed using the CellTiter-Glo^®^ Luminescent Cell Viability Assay (Promega, Charbonnières-les-Bains, France) that determine the number of viable cells based on the quantitation of adenosine triphosphate (ATP). The luminescent signal produced by the luciferase reaction, reflecting the presence of metabolically active cells, was read using a multiplate reader (TECAN, Lyon, France). The results were obtained by calculating the percentage of viability obtained by calculating the ratio of the luminescence in treated samples and the luminescence in non-treated samples.

### 5.4. Statistical Analysis

The reported values on viability are expressed as the means ± the standard error of the mean (SEM) of at least three biological replicates, with duplicate wells for each dose. The IC_50_ value, the dose of each toxin leading to 50% viability, was determined using CompuSyn statistical software (CompuSyn Version-1 Inc. Paramus, NJ, USA). Significant differences between groups were analyzed using the Bonferroni multiple comparison test in GraphPad (GraphPad Prism 4 La Jolla, CA, USA).

## Figures and Tables

**Table 1:** Top 60 emerging and regulated mycotoxins co-occurring with DON in finished pig feed.

	Metabolites	Occurrence *n* (%)	Co-occurrence with DON *n* (%)	Contamination Level (µg/kg Feed)			Correlation (DON and Other Mycotoxins)	
				P25	P50	P75	Coefficient	*p*-Value
1	Deoxynivalenol	463 (88%)	463 (100%)	111	206	389	1.00	NA
2	Culmorin	492 (94%)	458 (99%)	38	107	247	0.50	0.00
3	Zearalenone	502 (96%)	449 (97%)	9	18	46	0.64	0.00
4	Brevianamide F	500 (95%)	446 (96%)	17	28	45	0.17	0.00
5	Cyclo-(L-Pro-L-Tyr)	494 (94%)	434 (94%)	117	196	371	0.14	0.00
6	Enniatin B	479 (91%)	430 (93%)	9	32	83	0.02	0.61
7	Enniatin B1	481 (92%)	430 (93%)	10	37	87	0.04	0.39
8	Asperglaucide	470 (90%)	419 (90%)	18	38	94	-0.11	0.02
9	Emodin	472 (90%)	418 (90%)	3	5	10	-0.01	0.91
10	Aurofusarin	464 (89%)	417 (90%)	87	211	548	0.59	0.00
11	Moniliformin	469 (90%)	416 (90%)	7	17	45	0.11	0.03
12	Beauvericin	464 (89%)	411 (89%)	4	7	13	0.32	0.00
13	Enniatin A1	459 (88%)	411 (89%)	5	16	33	0.09	0.08
14	3-Nitropropion acid	455 (87%)	407 (88%)	3	6	10	-0.03	0.57
15	Tryptophol	454 (87%)	407 (88%)	119	197	319	0.10	0.04
16	15-Hydroxyculmorin	429 (82%)	391 (84%)	76	142	277	0.79	0.00
17	Equisetin	424 (81%)	386 (83%)	5	10	23	0.01	0.80
18	Infectopyron	409 (78%)	366 (79%)	108	263	449	-0.16	0.00
19	DON-3 Glucoside	380 (73%)	362 (78%)	10	21	47	0.79	0.00
20	Neoechinulin A	407 (78%)	357 (77%)	10	19	42	0.06	0.22
21	Tenuazonic-acid	384 (73%)	347 (75%)	53	90	182	0.04	0.49
22	Alternariol	366 (70%)	333 (72%)	2	4	9	0.01	0.79
23	Rugulusovin	373 (71%)	332 (72%)	4	7	14	0.15	0.01
24	Tentoxin	342 (65%)	319 (69%)	2	3	6	-0.03	0.56
25	Apicidin	341 (65%)	310 (67%)	3	7	11	-0.13	0.02
26	Fumonisin B1	332 (63%)	304 (66%)	26	70	254	0.14	0.02
27	Nivalenol	315 (60%)	296 (64%)	10	24	57	0.14	0.02
28	Cyclo-(L-Pro-L-Val)	337 (64%)	286 (62%)	85	137	246	0.15	0.01
29	Epiequisetin	307 (59%)	285 (62%)	2	4	7	0.05	0.42
30	Citreorosein	317 (60%)	283 (61%)	3	5	8	0.12	0.05
31	Enniatin A	306 (58%)	282 (61%)	2	3	5	-0.01	0.81
32	Alternariolmethylether	307 (59%)	275 (59%)	2	3	5	0.09	0.14
33	Altersetin	301 (57%)	275 (59%)	13	29	76	0.18	0.00
34	Asperphenamate	311 (59%)	269 (58%)	5	11	27	-0.02	0.75
35	Lotaustralin	288 (55%)	257 (56%)	15	30	85	-0.10	0.13
36	Butenolid	253 (48%)	242 (52%)	22	37	70	0.32	0.00
37	Kojic acid	262 (50%)	241 (52%)	43	74	148	-0.06	0.39
38	Enniatin B2	258 (49%)	238 (51%)	2	3	5	-0.01	0.84
39	Fumonisin B2	258 (49%)	237 (51%)	19	50	143	0.16	0.01
40	Zearalenone Sulfate	236 (45%)	222 (48%)	10	25	53	0.25	0.00
41	Antibiotic Y	233 (44%)	215 (46%)	40	111	402	-0.02	0.75
42	T2 Toxin	235 (45%)	209 (45%)	2	4	9	0.12	0.07
43	Macrosporin	219 (42%)	202 (44%)	2	3	8	0.02	0.76
44	N-Benzoyl-Phenylalanine	220 (42%)	191 (41%)	3	5	11	-0.02	0.82
45	Flavoglaucin	206 (39%)	175 (38%)	7	16	34	0.05	0.51
46	Curvularin	196 (37%)	171 (37%)	2	4	8	-0.09	0.27
47	Questiomycin A	178 (34%)	162 (35%)	4	10	20	0.22	0.01
48	Rubellin D	179 (34%)	161 (35%)	4	8	18	0.10	0.21
50	Bikaverin	171 (33%)	153 (33%)	10	25	56	0.27	0.00
50	Fusarinolic-acid	157 (30%)	153 (33%)	47	130	320	0.3	0.00
51	Fumonisin B4	165 (31%)	149 (32%)	11	23	68	0.2	0.03
52	Cytochalasin J	170 (32%)	146 (32%)	13	29	63	0.1	0.46
53	Ergometrine	152 (29%)	145 (31%)	6	11	24	0.0	0.57
54	Ergocristine	151 (29%)	143 (31%)	2	5	13	0.2	0.02
55	Fumonisin B3	154 (29%)	136 (29%)	24	48	103	0.1	0.15
56	HT2-toxin	149 (28%)	134 (29%)	13	20	30	0.2	0.01
57	Monocerin	144 (27%)	133 (29%)	1	2	3	0.2	0.02
58	Chrysogin	136 (26%)	126 (27%)	7	12	17	0.4	0.00
59	Ergosin	128 (24%)	123 (27%)	3	6	13	-0.1	0.39
60	5-Hydroxyculmorin	121 (23%)	117 (25%)	107	170	304	0.7	0.00

The 60 mycotoxins found in more than 20% of the 524 samples of finished pig feed. Their concentrations in the three quartiles (P25, P50 and P75) are expressed in µg/kg of feed. The correlation of their concentration with DON and the associated P-value was calculated using Spearman’s rank correlation coefficient.

**Table 2:** IC_50_ values of the selected emerging mycotoxins on IPEC-1 cells.

Metabolites	Abbreviation	IC_50_ (µM)	Toxicity
Brevianamide F	BRV-F	Non-Toxic	Non-toxic
Cyclo-(L-Pro-L-Tyr)	Cyclo	Non-Toxic	
Tryptophol	TRPT	Non-Toxic	
Aurofusarin	AFN	19.1 ± 3.4	Moderately toxic
Emodin	EMO	19.0 ± 0.7	
Enniatin B1	ENN-B1	13.5 ± 2.5	
Enniatin B	ENN-B	4.4 ± 0.9	Highly toxic
Beauvericin	BEA	4.3 ± 1.8	
Deoxynivalenol	DON	3.2 ± 0.7	
Enniatin A1	ENN-A1	1.6 ± 0.3	
Apicidin	API	1.5 ± 0.5	

Data are the mean ± the standard error of the mean (SEM) of three biological replicates.

## Supplementary Materials

The following are available online at https://www.mdpi.com/2072-6651/11/12/727/s1, Figure S1: Worldwide contamination of emerging mycotoxins. Figure S2: Abundance of emerging mycotoxins in finished pig feed. Figure S3: Scatter plot of the correlation between DON and 10 selected emerging mycotoxins. Figure S4: Dose-effect curves of the individual toxicity of DON and 9 selected fungal emerging mycotoxins Table S1: Concentration of the metabolites in 3 quartiles (P25, P50 and P75) and ratios between the concentration of DON and 10 selected emerging mycotoxins. Figure S5: LC-ESI(+)-MRM chromatograms of one pig feed sample. Figure S6: Overlay of the extracted ion chromatograms of both quantifier as well as qualifier of the 11 investigated compounds in one pig feed sample.

## References

[1] Bennett J.W., Klich M. (2003). Mycotoxins. Clin. Microbiol. Rev..

[2] European Commission (2006). Commission Recommendation of 17 August 2006 on the presence of deoxynivalenol, zearalenone, ochratoxin A, T-2 and HT-2 and fumonisins in products intended for animal feeding. Off. J. Eur. Union.

[3] European Commission (2013). Commission Recommendation of 27 March 2013 on the presence of T-2 and HT-2 toxin in cereals and cereal productsText with EEA relevance. Off. J. Eur. Union.

[4] Berthiller F., Crews C., Dall’Asta C., Saeger S.D., Haesaert G., Karlovsky P., Oswald I.P., Seefelder W., Speijers G., Stroka J. (2013). Masked mycotoxins: A review. Mol. Nutr. Food Res..

[5] Malachová A., Sulyok M., Beltrán E., Berthiller F., Krska R. (2014). Optimization and validation of a quantitative liquid chromatography–tandem mass spectrometric method covering 295 bacterial and fungal metabolites including all regulated mycotoxins in four model food matrices. J. Chromatogr. A.

[6] Gruber-Dorninger C., Novak B., Nagl V., Berthiller F. (2016). Emerging Mycotoxins: Beyond Traditionally Determined Food Contaminants. J. Agric. Food Chem..

[7] Rychlik M., Humpf H.-U., Marko D., Dänicke S., Mally A., Berthiller F., Klaffke H., Lorenz N. (2014). Proposal of a comprehensive definition of modified and other forms of mycotoxins including “masked” mycotoxins. Mycotoxin Res..

[8] Eskola M., Kos G., Elliott C.T., Hajšlová J., Mayar S., Krska R. Worldwide contamination of food-crops with mycotoxins: Validity of the widely cited ‘FAO estimate’ of 25%. Crit. Rev. Food Sci. Nutr..

[9] Schatzmayr G., Streit E. (2013). Global occurrence of mycotoxins in the food and feed chain: Facts and figures. World Mycotoxin J..

[10] Streit E., Schatzmayr G., Tassis P., Tzika E., Marin D., Taranu I., Tabuc C., Nicolau A., Aprodu I., Puel O. (2012). Current Situation of Mycotoxin Contamination and Co-occurrence in Animal Feed—Focus on Europe. Toxins.

[11] Knutsen H.K., Alexander J., Barregard L., Bignami M., Bruschweiler B., Ceccatelli S., Cottrill B., Dinovi M., Grasl-Kraupp B., Hogstrand C. (2017). Risks to human and animal health related to the presence of deoxynivalenol and its acetylated and modified forms in food and feed. EFSA J..

[12] IARC (1993). Toxins derived from *Fusarium* graminearum, F. culmorum and F. crookwellense: Zearalenone, deoxynivalenol, nivalenol and fusarenone X. IARC Monogr. Eval. Carcinog. Risks Hum..

[13] Charmley L., Trenholm H. RG-8 Regulatory Guidance:Contaminants in Feed (Formerly RG-1, Chapter 7). https://www.inspection.gc.ca/animals/feeds/regulatory-guidance/rg-8/eng/1347383943203/1347384015909?chap=1#s1c1.

[14] Pestka J.J. (2010). Deoxynivalenol: Mechanisms of action, human exposure, and toxicological relevance. Arch. Toxicol..

[15] Pinton P., Oswald I. (2014). Effect of Deoxynivalenol and Other Type B Trichothecenes on the Intestine: A Review. Toxins.

[16] Payros D., Alassane-Kpembi I., Pierron A., Loiseau N., Pinton P., Oswald I.P. (2016). Toxicology of deoxynivalenol and its acetylated and modified forms. Arch. Toxicol..

[17] Pierron A., Mimoun S., Murate L.S., Loiseau N., Lippi Y., Bracarense A.-P.F.L., Schatzmayr G., He J.W., Zhou T., Moll W.-D. (2016). Microbial biotransformation of DON: Molecular basis for reduced toxicity. Sci. Rep..

[18] Jajić I., Dudaš T., Krstović S., Krska R., Sulyok M., Bagi F., Savić Z., Guljaš D., Stankov A. (2019). Emerging *Fusarium* Mycotoxins Fusaproliferin, Beauvericin, Enniatins, and Moniliformin in Serbian Maize. Toxins.

[19] EFSA (2014). Scientific Opinion on the risks to human and animal health related to the presence of beauvericin and enniatins in food and feed: Beauvericin and enniatins in food and feed. EFSA J..

[20] Novak B., Rainer V., Sulyok M., Haltrich D., Schatzmayr G., Mayer E. (2019). Twenty-Eight Fungal Secondary Metabolites Detected in Pig Feed Samples: Their Occurrence, Relevance and Cytotoxic Effects In Vitro. Toxins.

[21] Abdallah M.F., Girgin G., Baydar T., Krska R., Sulyok M. (2017). Occurrence of multiple mycotoxins and other fungal metabolites in animal feed and maize samples from Egypt using LC-MS/MS: Toxic fungal and bacterial metabolites in feed and maize from Egypt. J. Sci. Food Agric..

[22] Streit E., Schwab C., Sulyok M., Naehrer K., Krska R., Schatzmayr G. (2013). Multi-Mycotoxin Screening Reveals the Occurrence of 139 Different Secondary Metabolites in Feed and Feed Ingredients. Toxins.

[23] Anjorin S.T., Fapohunda S., Sulyok M., Krska R. (2016). Natural Co-occurrence of Emerging and Minor Mycotoxins on Maize Grains from Abuja, Nigeria. Ann. Agric. Environ. Sci..

[24] Alassane-Kpembi I., Schatzmayr G., Taranu I., Marin D., Puel O., Oswald I.P. (2017). Mycotoxins co-contamination: Methodological aspects and biological relevance of combined toxicity studies. Crit. Rev. Food Sci. Nutr..

[25] Fremy J.-M., Alassane-Kpembi I., Oswald I.P., Cottrill B., Van Egmond H.P. (2019). A review on combined effects of moniliformin and co-occurring *Fusarium* toxins in farm animals. World Mycotoxin J..

[26] Assunção R., Silva M.J., Alvito P. (2016). Challenges in risk assessment of multiple mycotoxins in food. World Mycotoxin J..

[27] More S.J., Bampidis V., Benford D., Bennekou S.H., Bragard C., Halldorsson T.I., Herna A.F., Koutsoumanis K., Naegeli H., Schlatter J.R. (2019). Guidance on harmonised methodologies for human health, animal health and ecological risk assessment of combined exposure to multiple chemicals. EFSA J..

[28] Meek B., Roobis A.R., Crofton K.M., Heinemeyer G., Raaij M.V., Vickers C. (2011). Risk assessment of combined exposure to multiple chemicals: A WHO/IPCS framework. Regul. Toxicol. Pharmacol..

[29] Kortenkamp A., Faust M. (2018). Regulate to reduce chemical mixture risk. Science.

[30] Ghareeb K., Awad W.A., Böhm J., Zebeli Q. (2015). Impacts of the feed contaminant deoxynivalenol on the intestine of monogastric animals: Poultry and swine. J. Appl. Toxicol..

[31] Alassane-Kpembi I., Kolf-Clauw M., Gauthier T., Abrami R., Abiola F.A., Oswald I.P., Puel O. (2013). New insights into mycotoxin mixtures: The toxicity of low doses of Type B trichothecenes on intestinal epithelial cells is synergistic. Toxicol. Appl. Pharmacol..

[32] Alassane-Kpembi I., Puel O., Oswald I.P. (2015). Toxicological interactions between the mycotoxins deoxynivalenol, nivalenol and their acetylated derivatives in intestinal epithelial cells. Arch. Toxicol..

[33] Alassane-Kpembi I., Puel O., Pinton P., Cossalter A.-M., Chou T.-C., Oswald I. (2017). Co-exposure to low doses of the food contaminants deoxynivalenol and nivalenol has a synergistic inflammatory effect on intestinal explants. Arch. Toxicol..

[34] Kolf-Clauw M., Sassahara M., Lucioli J., Rubira-Gerez J., Alassane-Kpembi I., Lyazhri F., Borin C., Oswald I.P. (2013). The emerging mycotoxin, enniatin B1, down-modulates the gastrointestinal toxicity of T-2 toxin in vitro on intestinal epithelial cells and ex vivo on intestinal explants. Arch. Toxicol..

[35] Escrivá L., Jennen D., Caiment F., Manyes L. (2018). Transcriptomic study of the toxic mechanism triggered by beauvericin in Jurkat cells. Toxicol. Lett..

[36] Pierron A., Alassane-Kpembi I., Oswald I.P. (2016). Impact of mycotoxin on immune response and consequences for pig health. Anim. Nutr..

[37] Pinton P., Braicu C., Nougayrede J.-P., Laffitte J., Taranu I., Oswald I.P. (2010). Deoxynivalenol Impairs Porcine Intestinal Barrier Function and Decreases the Protein Expression of Claudin-4 through a Mitogen-Activated Protein Kinase-Dependent Mechanism. J. Nutr..

[38] Council for Agricultural Science and Technology (2003). Mycotoxins: Risks in Plant, Animal, and Human Systems.

[39] Bauden M., Tassidis H., Ansari D. (2015). In vitro cytotoxicity evaluation of HDAC inhibitor Apicidin in pancreatic carcinoma cells subsequent time and dose dependent treatment. Toxicol. Lett..

[40] Glenn A.E., Bacon C.W., Price R., Hanlin R.T. (1996). Molecular phylogeny of *Acremonium* and its taxonomic implications. Mycologia.

[41] Kosalec I., Ramić S., Jelić D., Antolović R., Pepeljnjak S., Kopjar N. (2011). Assessment of Tryptophol Genotoxicity in Four Cell Lines In Vitro: A Pilot Study with Alkaline Comet Assay. Arch. Ind. Hyg. Toxicol..

[42] Muto A., Hori M., Sasaki Y., Saitoh A., Yasuda I., Maekawa T., Uchida T., Asakura K., Nakazato T., Kaneda T. (2007). Emodin has a cytotoxic activity against human multiple myeloma as a Janus-activated kinase 2 inhibitor. Mol. Cancer Ther..

[43] Meca G., Font G., Ruiz M.J. (2011). Comparative cytotoxicity study of enniatins A, A1, A2, B, B1, B4 and J3 on Caco-2 cells, Hep-G2 and HT-29. Food Chem. Toxicol..

[44] Fraeyman S., Meyer E., Devreese M., Antonissen G., Demeyere K., Haesebrouck F., Croubels S. (2018). Comparative in vitro cytotoxicity of the emerging *Fusarium* mycotoxins beauvericin and enniatins to porcine intestinal epithelial cells. Food Chem. Toxicol..

[45] Taranu I., Marin D.E., Burlacu R., Pinton P., Damian V., Oswald I.P. (2010). Comparative aspects of *in vitro* proliferation of human and porcine lymphocytes exposed to mycotoxins. Arch. Anim. Nutr..

[46] Gruber-Dorninger C., Jenkins T., Schatzmayr G. (2019). Global Mycotoxin Occurrence in Feed: A Ten-Year Survey. Toxins.

[47] Alkadri D., Rubert J., Prodi A., Pisi A., Mañes J., Soler C. (2014). Natural co-occurrence of mycotoxins in wheat grains from Italy and Syria. Food Chem..

[48] Sulyok M., Stadler D., Steiner D., Krska R. Validation of an LC-MS/MS based dilute-and-shoot approach for the quantification of >500 mycotoxins and other secondary metabolites in food crops: Challenges and solutions.

[49] Blandino M., Scarpino V., Sulyok M., Krska R., Reyneri A. (2017). Effect of agronomic programmes with different susceptibility to deoxynivalenol risk on emerging contamination in winter wheat. Eur. J. Agron..

[50] Prosperini A., Font G., Ruiz M.J. (2014). Interaction effects of *Fusarium* enniatins (A, A1, B and B1) combinations on in vitro cytotoxicity of Caco-2 cells. Toxicol. Vitr..

[51] Fernández-Blanco C., Font G., Ruiz M.-J. (2016). Interaction effects of enniatin B, deoxinivalenol and alternariol in Caco-2 cells. Toxicol. Lett..

[52] Ireland J.J., Roberts R.M., Palmer G.H., Bauman D.E., Bazer F.W. (2008). A commentary on domestic animals as dual-purpose models that benefit agricultural and biomedical research. Am. Soc. Anim. Sci..

[53] García G.R., Payros D., Pinton P., Dogi C.A., Laffitte J., Neves M., González Pereyra M.L., Cavaglieri L.R., Oswald I.P. (2018). Intestinal toxicity of deoxynivalenol is limited by Lactobacillus rhamnosus RC007 in pig jejunum explants. Arch. Toxicol..

[54] Pinton P., Nougayrède J.-P., Del Rio J.-C., Moreno C., Marin D.E., Ferrier L., Bracarense A.-P., Kolf-Clauw M., Oswald I.P. (2009). The food contaminant deoxynivalenol, decreases intestinal barrier permeability and reduces claudin expression. Toxicol. Appl. Pharmacol..

