# Supplementary material for: Co-Occurrence of DON and Emerging Mycotoxins in Worldwide Finished Pig Feed and Their Combined Toxicity in Intestinal Cells
Source: Toxins (Basel). 2019 Dec 11;11(12):727. doi: 10.3390/toxins11120727 (PMC6950336; doi:10.3390/toxins11120727)
Supplement: Supplementary file 1 [file toxins-11-00727-s001.pdf]

Co-occurrence of DON and Emerging Mycotoxins in Worldwide Finished Pig Feed and Their Combined Toxicity in Intestinal Cells

Abdullah Khan Khoshal, Barbara Novak, Pascal G.P. Martin, Timothy Jenkins, Manon Neves, Gerd Schatzmayr, Isabelle P. Oswald and Philippe Pinton

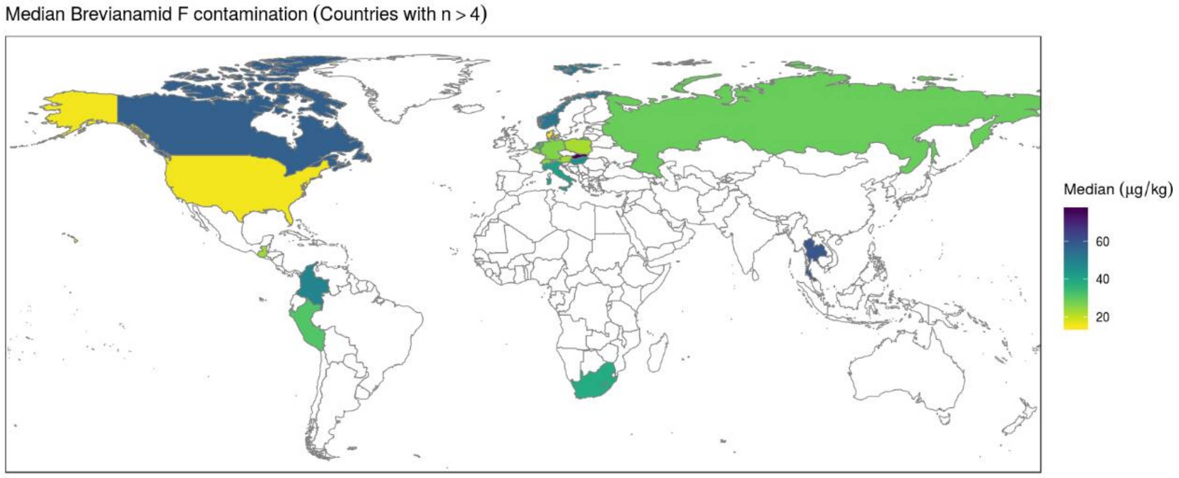

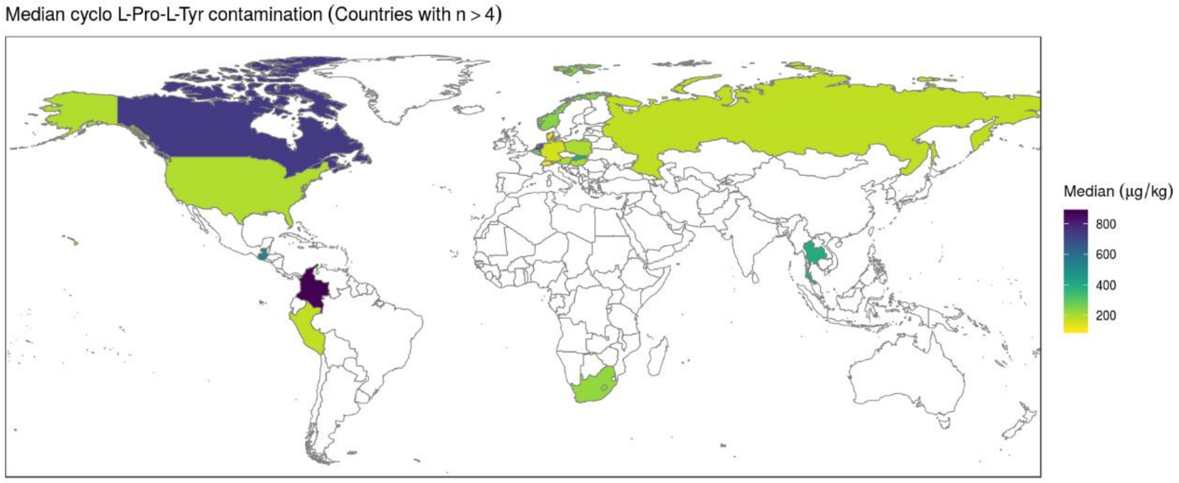

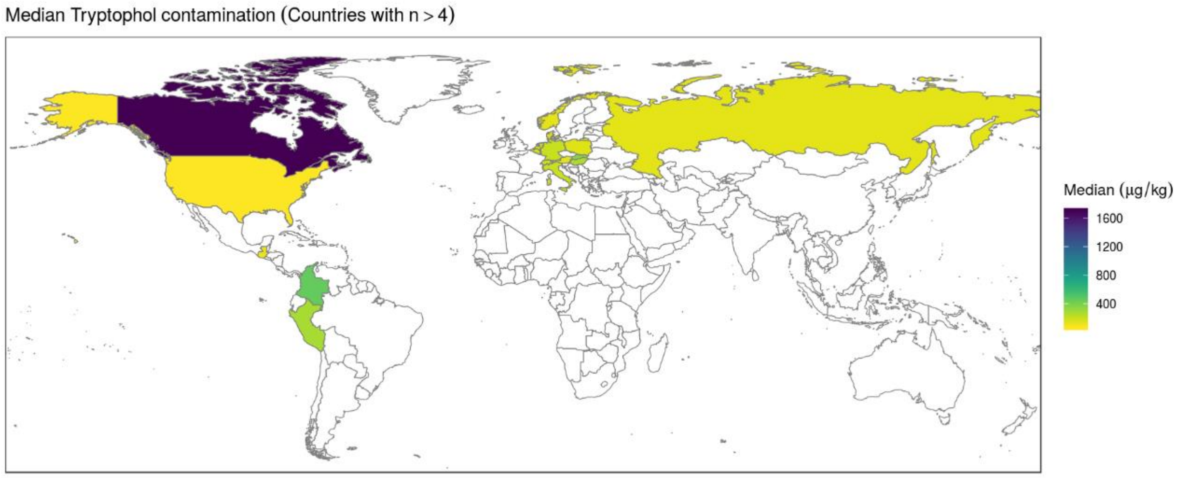

Median Aurofusarin contamination (Countries with n > 4)

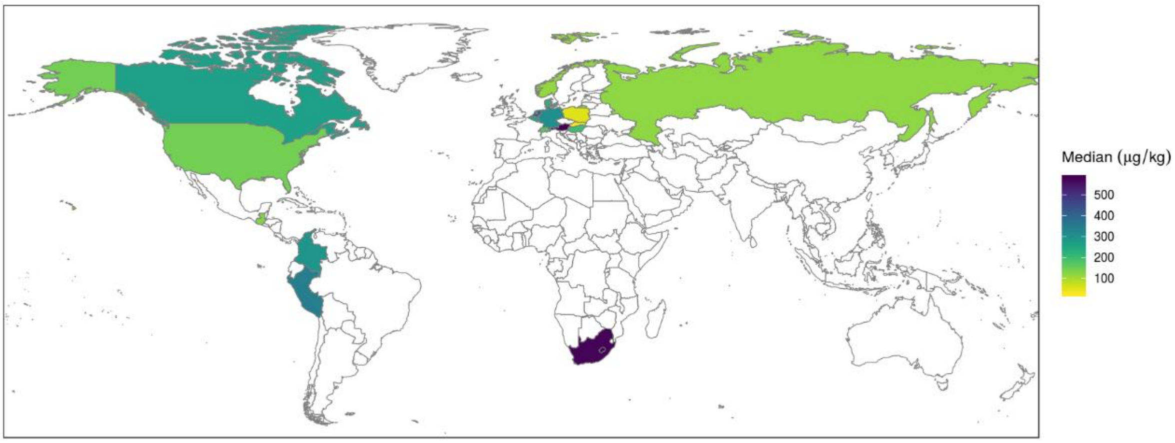

Median Emodin contamination (Countries with n > 4)

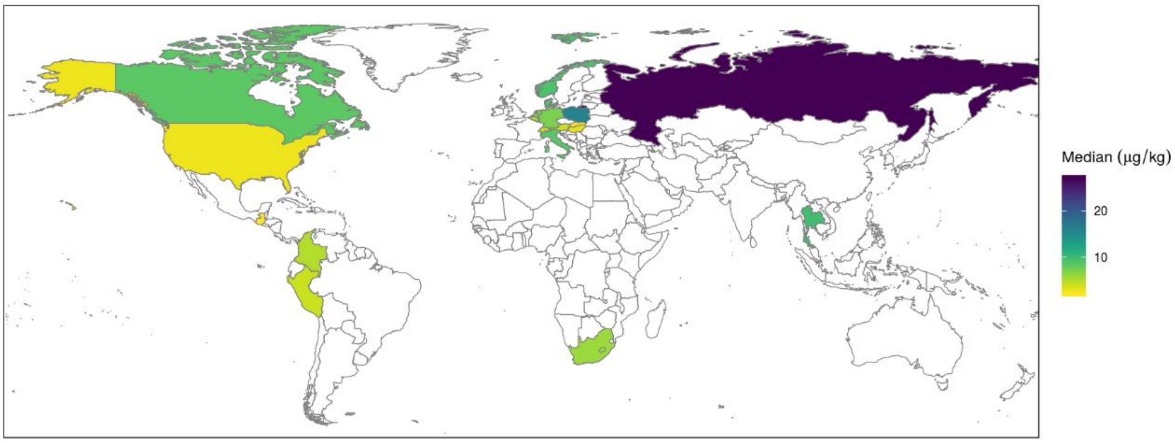

Median Enniatin B1 contamination (Countries with n > 4)

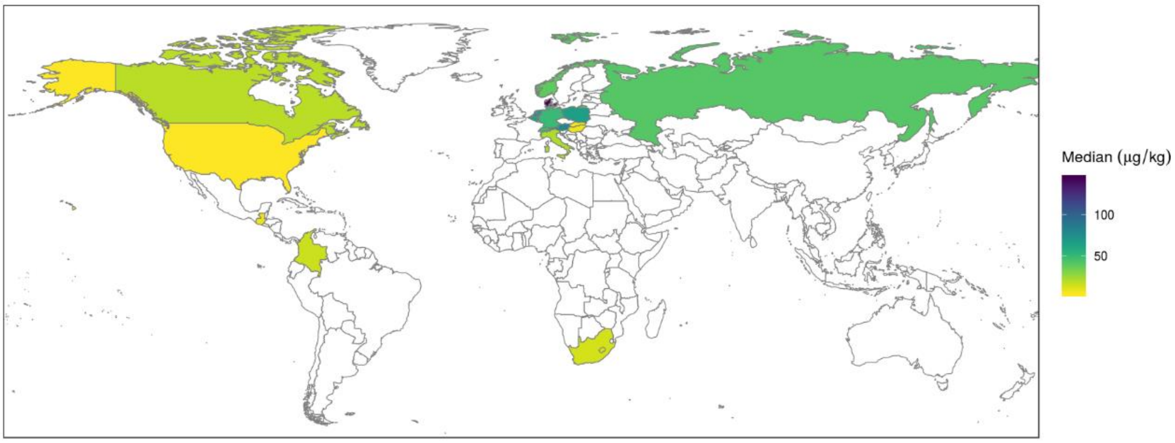

Median Enniatin B contamination (Countries with n > 4)

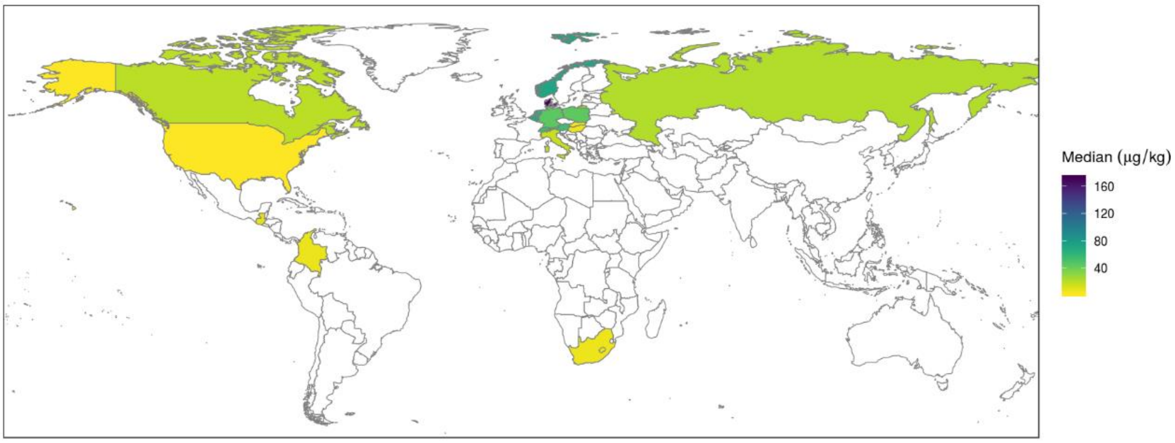

Median Beauvericin contamination (Countries with n > 4)

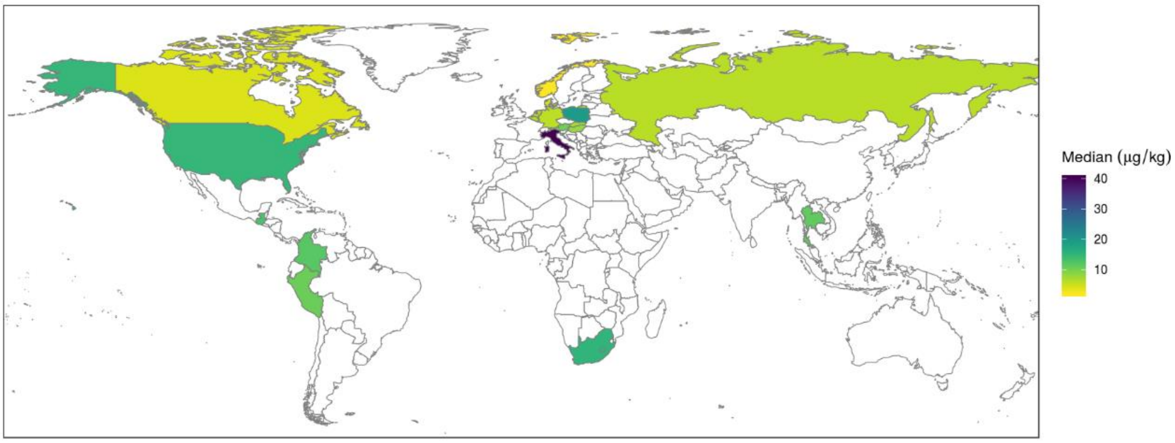

Median Enniatin A1 contamination (Countries with n > 4)

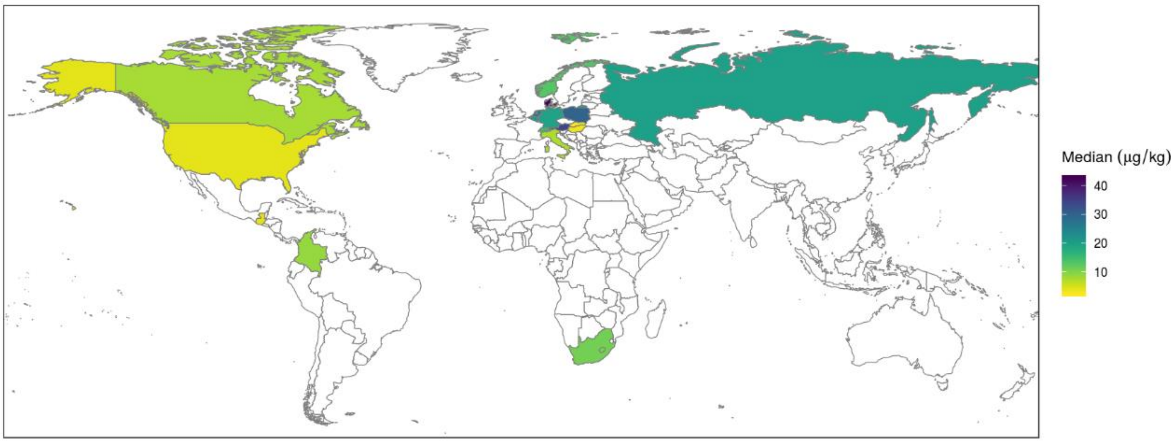

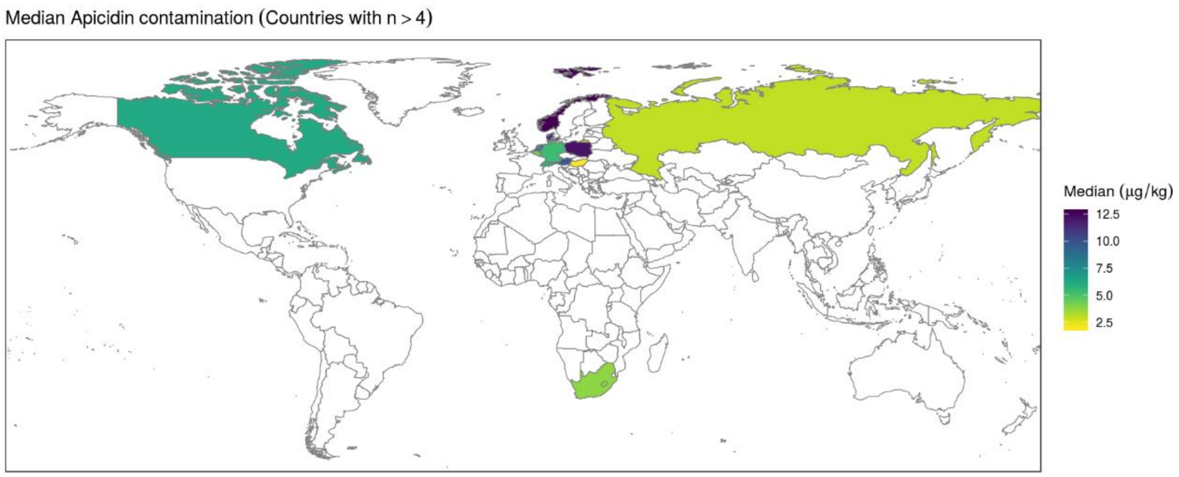

Figure S1. Worldwide contamination of BRV-F, Cyclo, TRPT, EMO, ENN-B1, AFN, ENN-A1, ENN-B, BEA and API.

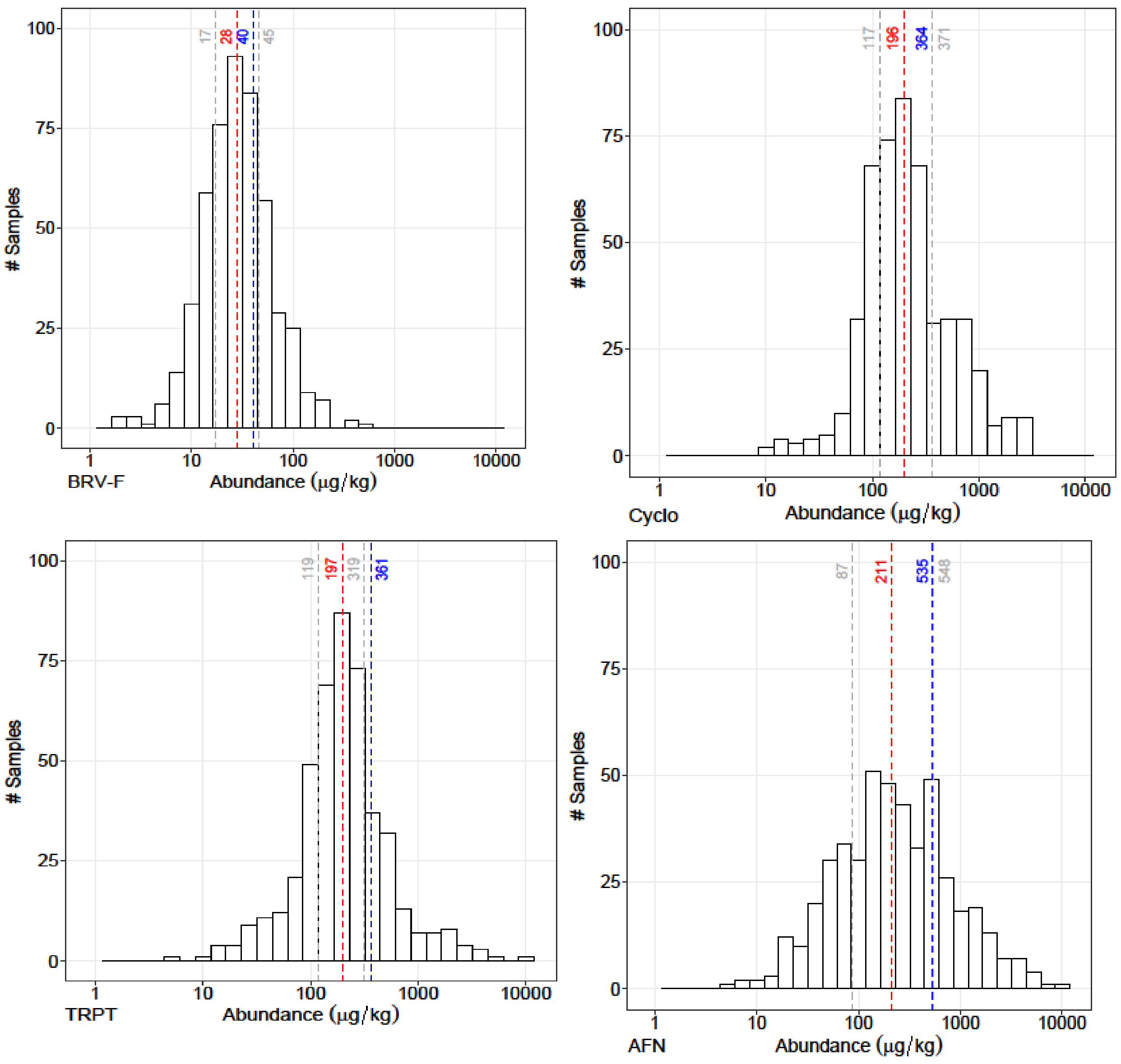

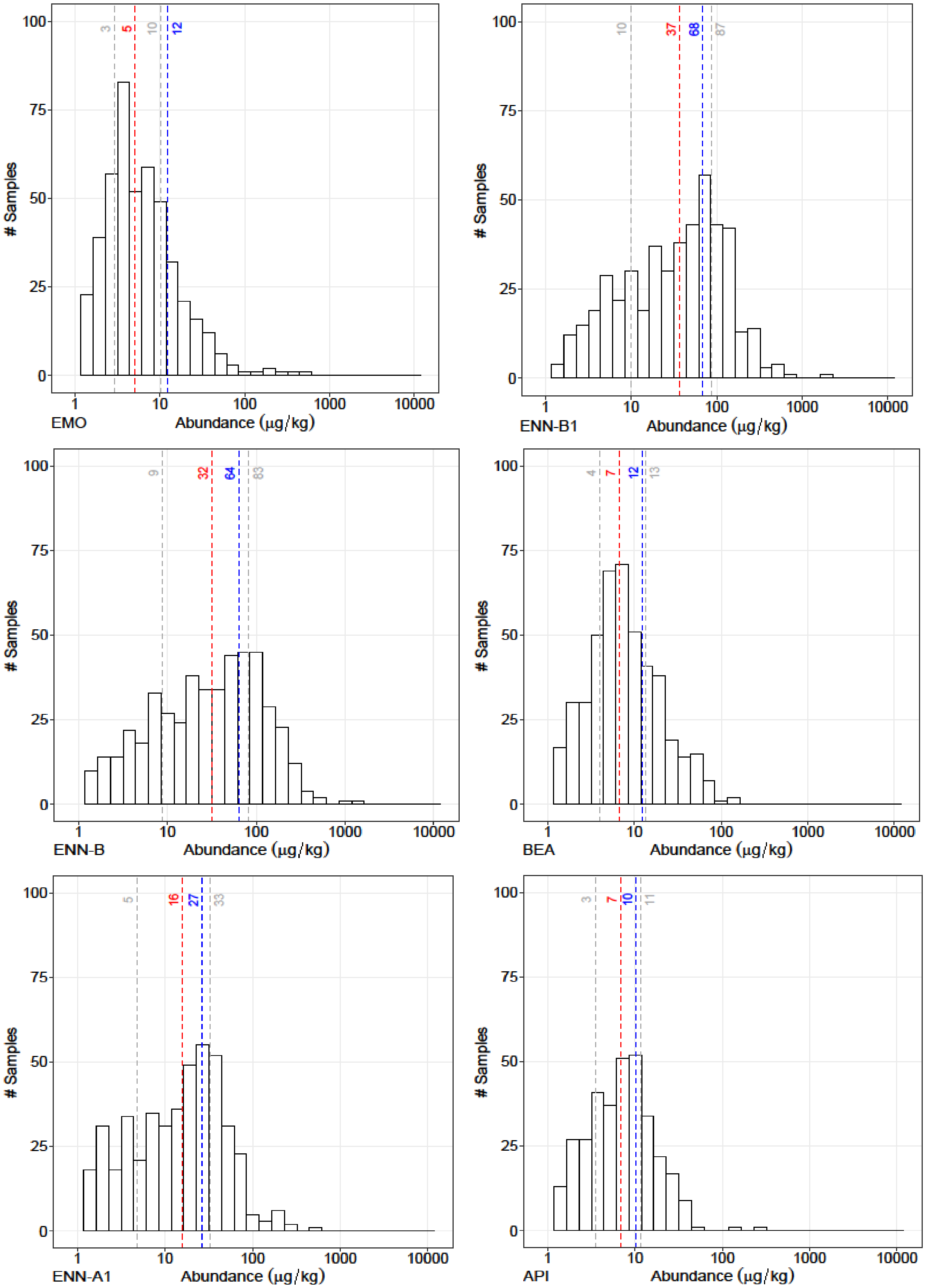

Figure S2. Abundance of emerging mycotoxins in pig feed (P25, median, mean, P75). X axis represents the distribution of the concentrations of fungal secondary metabolites (ppb). Y-axis describes the number of co-contaminated samples by DON and other emerging mycotoxins. Median concentrations

of AFN, TRPT and Cyclo were ≈ 200 ppb, median concentrations of ENN-B1, ENN-B, BRV-F and ENN-A1 were 15 -40 ppb, and median concentrations of API, BEA and EMO were 5-10 ppb.

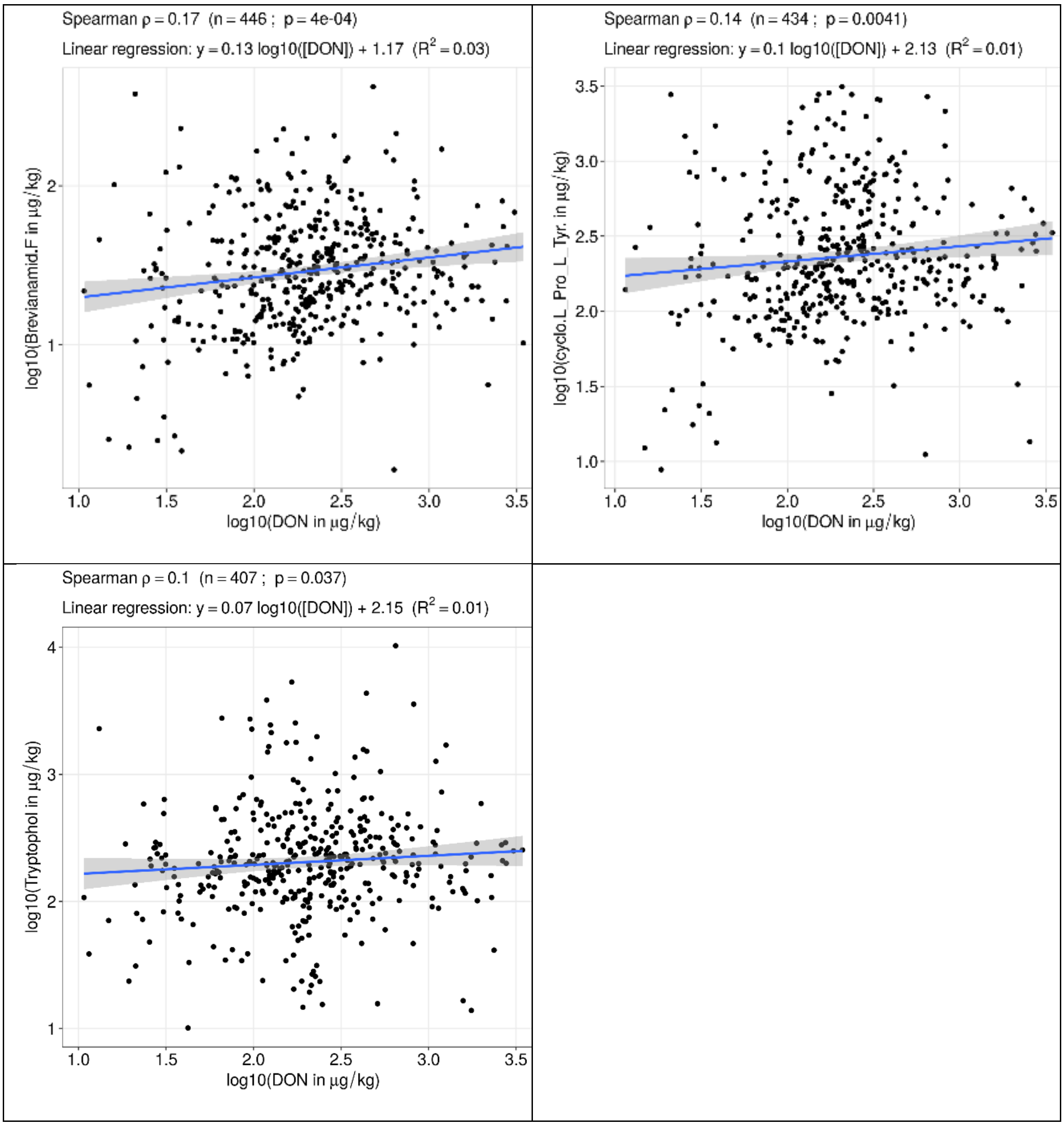

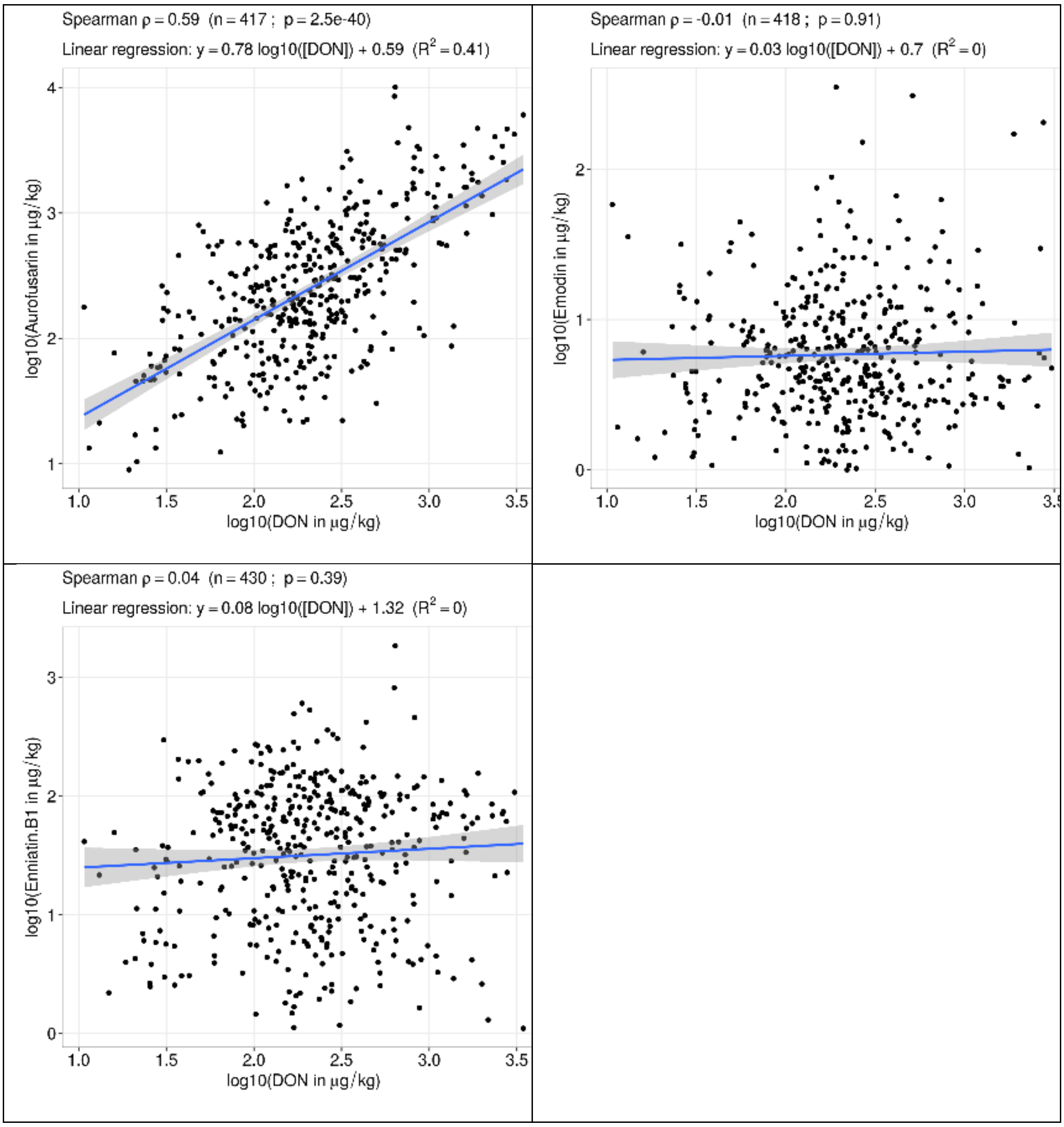
Spearman ρ = 0.59 (n = 417 ; p = 2.5e-40)
Linear regression: y = 0.78 log10([DON]) + 0.59 (R2 = 0.41)
log10(Aurofusarin in µg/kg)
log10(DON in µg/kg)
Spearman ρ = -0.01 (n = 418 ; p = 0.91)
Linear regression: y = 0.03 log10([DON]) + 0.7 (R2 = 0)
log10(Emodin in µg/kg)
log10(DON in µg/kg)
Spearman ρ = 0.04 (n = 430 ; p = 0.39)
Linear regression: y = 0.08 log10([DON]) + 1.32 (R2 = 0)
log10(Enniatin.B1 in µg/kg)
log10(DON in µg/kg)

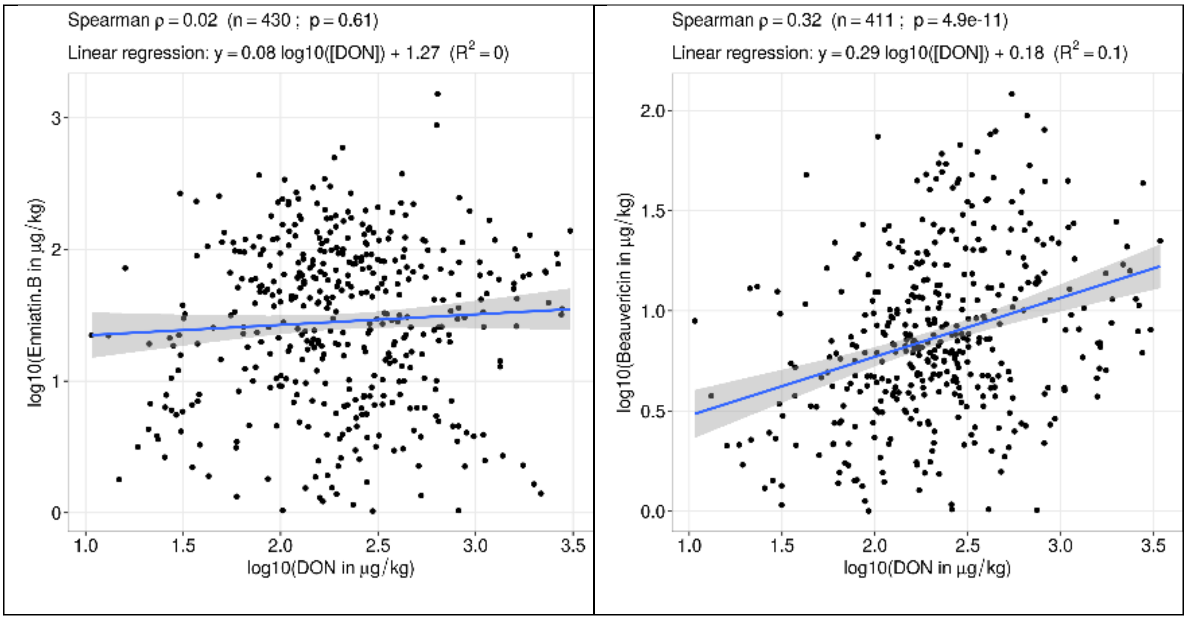
Spearman ρ = 0.02 (n = 430 ; p = 0.61)
Linear regression: y = 0.08 log10([DON]) + 1.27 (R2 = 0)
log10(Enniatin.B in µg/kg)
log10(DON in µg/kg)
Spearman ρ = 0.32 (n = 411 ; p = 4.9e-11)
Linear regression: y = 0.29 log10([DON]) + 0.18 (R2 = 0.1)
log10(Beauvericin in µg/kg)
log10(DON in µg/kg)

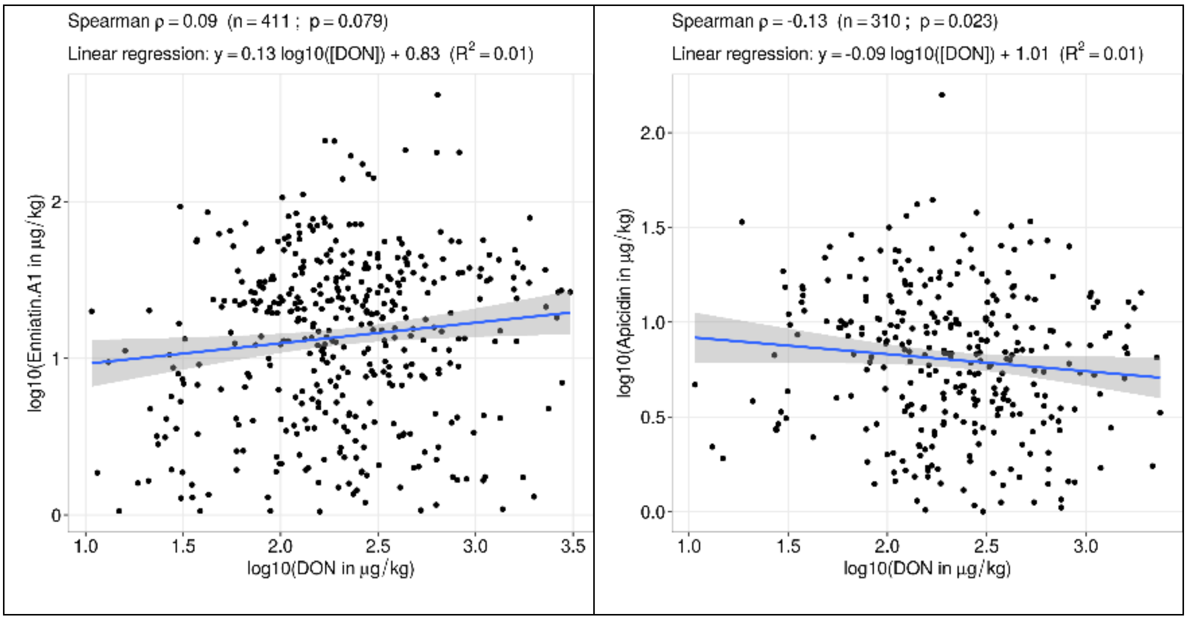

Figure S3. Scatter plot showing the correlation between the concentrations of BRV-F, Cyclo, TRPT, AFN, EMO, ENN-B1, ENN-B, BEA, DON, ENN-A1 and API in pig feed samples.

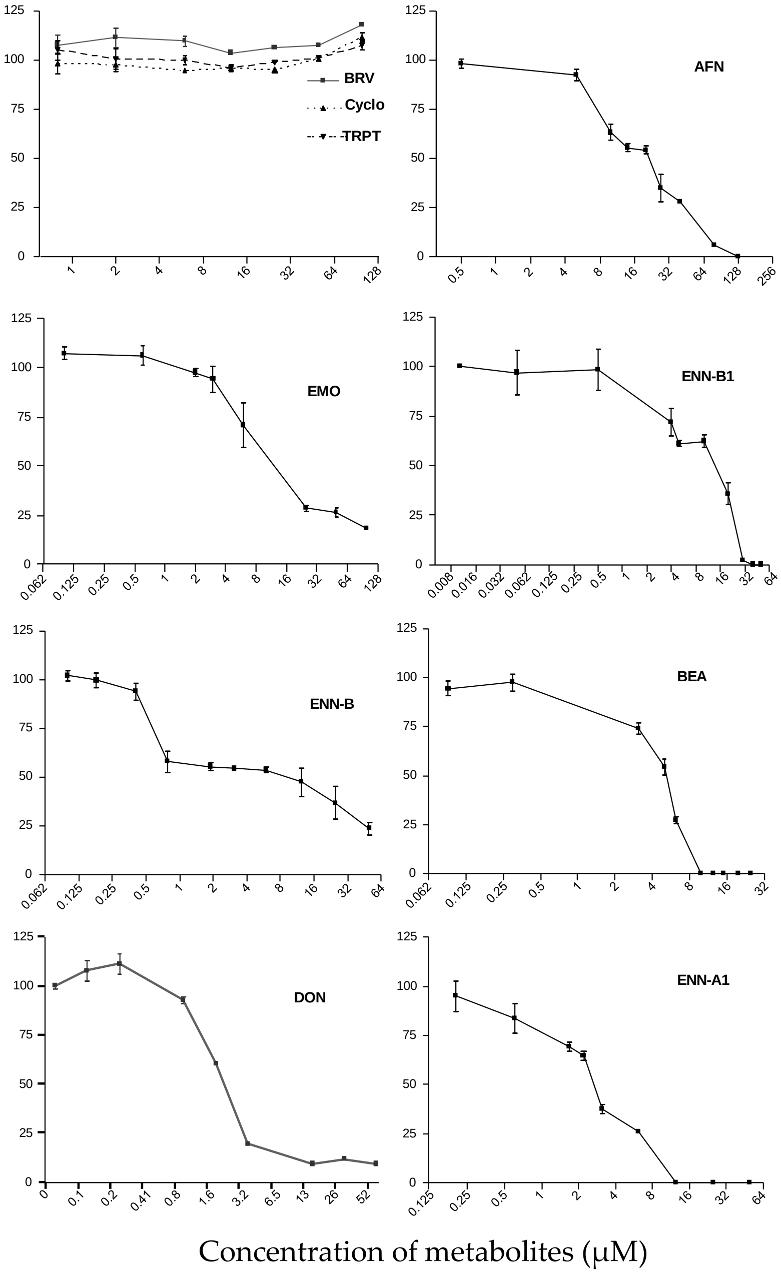

Figure S4. Dose effect curve of individual toxicity of 9 selected emerging mycotoxins (BRV-F, Cyclo, TRPT, AFN, EMO, ENN-B1, ENN-B, BEA, ENN-A1) and DON. Data are mean ± SEM of three biological replicates.

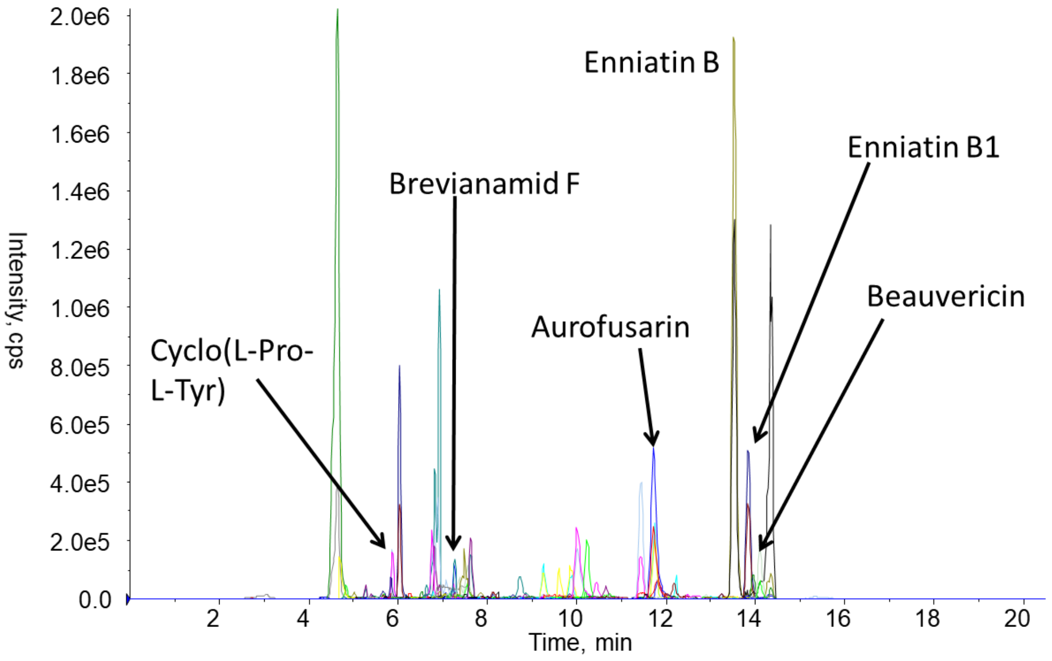

Figure S5a. LC-ESI(+)-MRM chromatogram (overlay of all acquired MRMs) of AT5-4153-009 (only compounds for toxicity tests are annotated).

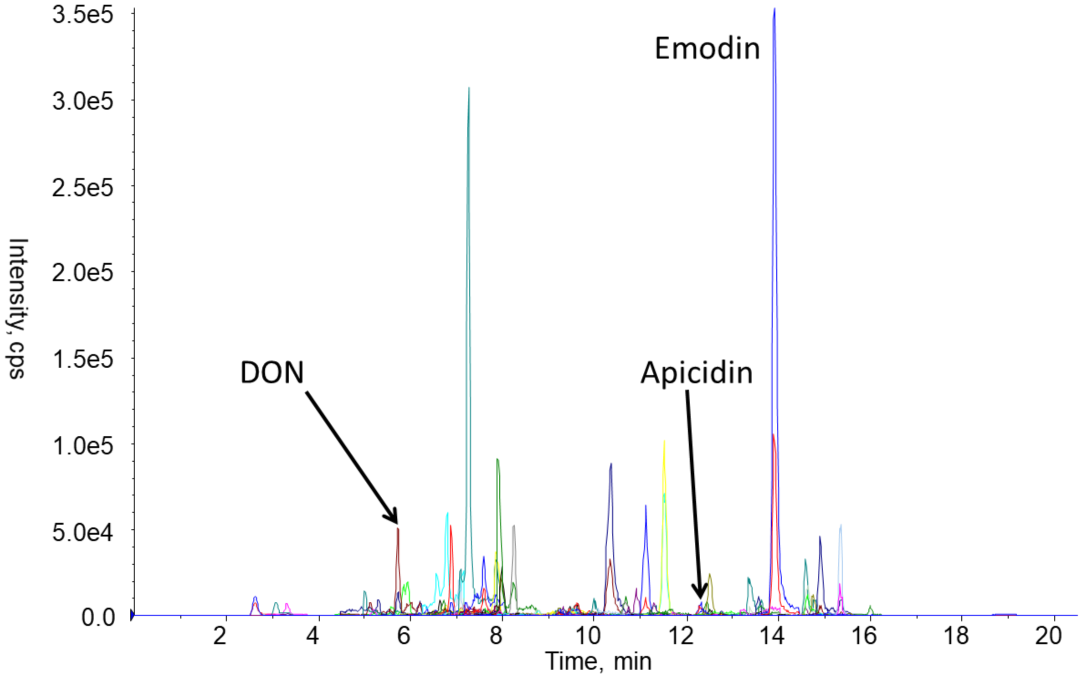

Figure S5b. LC-ESI(-)-MRM chromatogram (overlay of all acquired MRMs) of AT5-4153-009 (only compounds for toxicity tests are annotated).

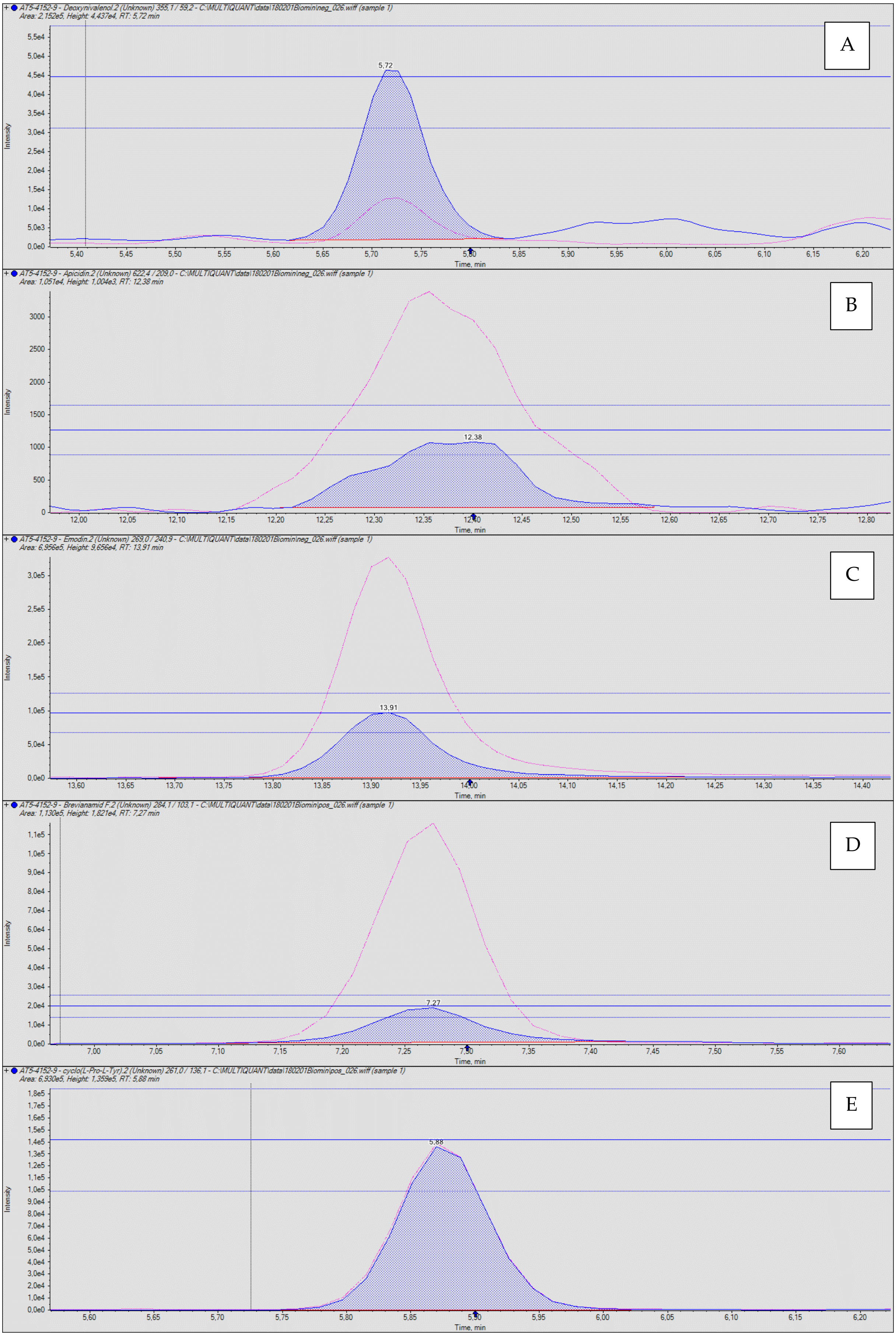
A
AT5-4152-9 - Deoxynivalenol.2 (Unknown) 355,1 / 59,2 - C:\MULTIQUANT\data\180201Biomin\neg_026.wiff (sample 1)
Area: 2,152e5, Height: 4,437e4, RT: 5,72 min
B
AT5-4152-9 - Apicidin.2 (Unknown) 622,4 / 209,0 - C:\MULTIQUANT\data\180201Biomin\neg_026.wiff (sample 1)
Area: 1,051e4, Height: 1,004e3, RT: 12,38 min
C
AT5-4152-9 - Emodin.2 (Unknown) 269,0 / 240,9 - C:\MULTIQUANT\data\180201Biomin\neg_026.wiff (sample 1)
Area: 6,956e5, Height: 9,656e4, RT: 13,91 min
D
AT5-4152-9 - Brevianamid F.2 (Unknown) 284,1 / 103,1 - C:\MULTIQUANT\data\180201Biomin\pos_026.wiff (sample 1)
Area: 1,130e5, Height: 1,821e4, RT: 7,27 min
E
AT5-4152-9 - cyclo(L-Pro-L-Tyr).2 (Unknown) 261,0 / 136,1 - C:\MULTIQUANT\data\180201Biomin\pos_026.wiff (sample 1)
Area: 6,930e5, Height: 1,359e5, RT: 5,88 min
Intensity
Time, min

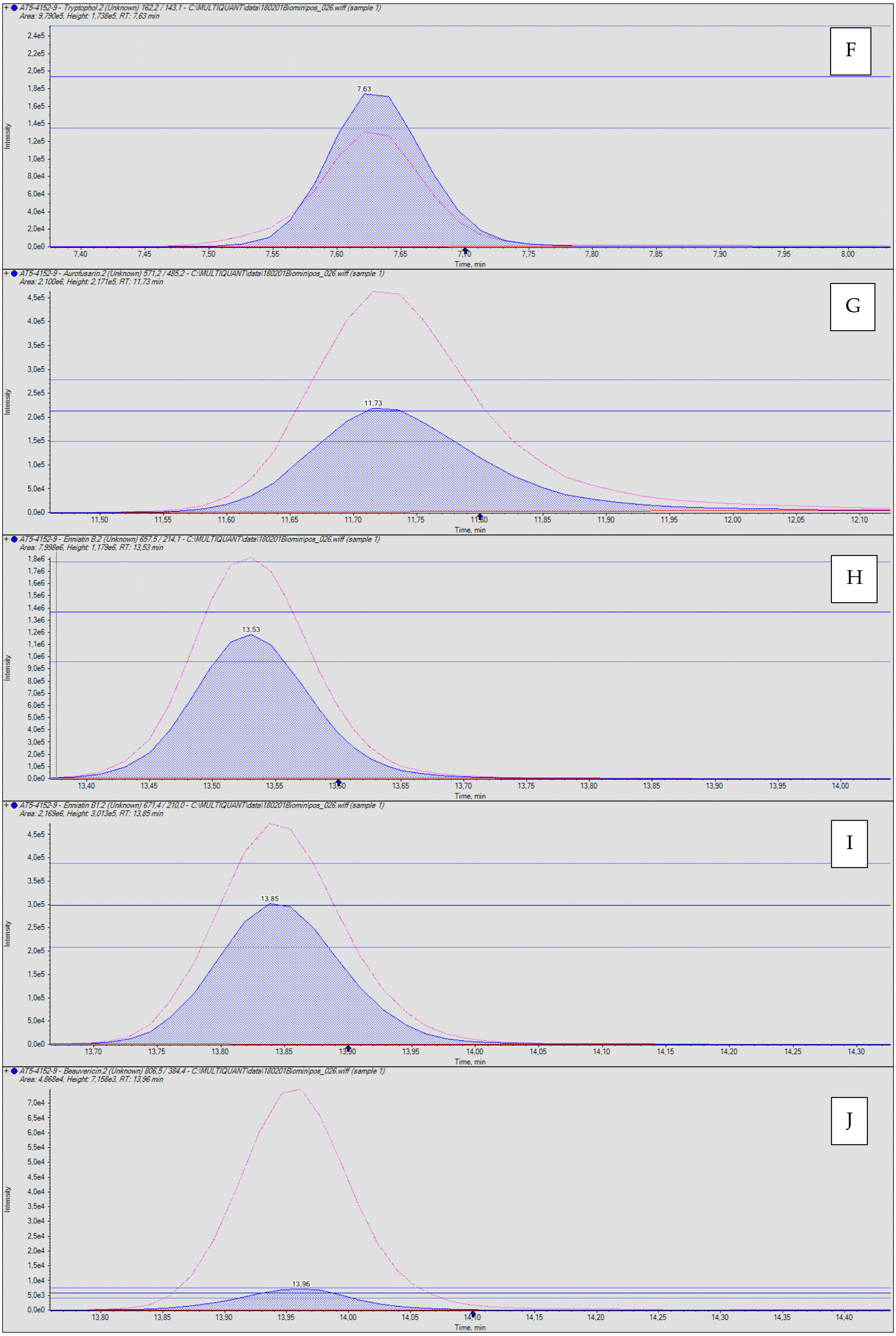
AT5-4152-9 - Tryptophol.2 (Unknown) 162,2 / 143,1 - C:\MULTIQUANT\data\180201Biomin\pos_026.wiff (sample 1)
Area: 9,790e5, Height: 1,738e5, RT: 7,63 min
F
AT5-4152-9 - Aurofusarin.2 (Unknown) 571,2 / 485,2 - C:\MULTIQUANT\data\180201Biomin\pos_026.wiff (sample 1)
Area: 2,100e6, Height: 2,171e5, RT: 11,73 min
G
AT5-4152-9 - Enniatin B.2 (Unknown) 657,5 / 214,1 - C:\MULTIQUANT\data\180201Biomin\pos_026.wiff (sample 1)
Area: 7,998e6, Height: 1,179e6, RT: 13,53 min
H
AT5-4152-9 - Enniatin B1.2 (Unknown) 671,4 / 210,0 - C:\MULTIQUANT\data\180201Biomin\pos_026.wiff (sample 1)
Area: 2,169e6, Height: 3,013e5, RT: 13,85 min
I
AT5-4152-9 - Beauvericin.2 (Unknown) 806,5 / 384,4 - C:\MULTIQUANT\data\180201Biomin\pos_026.wiff (sample 1)
Area: 4,868e4, Height: 7,158e3, RT: 13,96 min
J
Intensity
Time, min

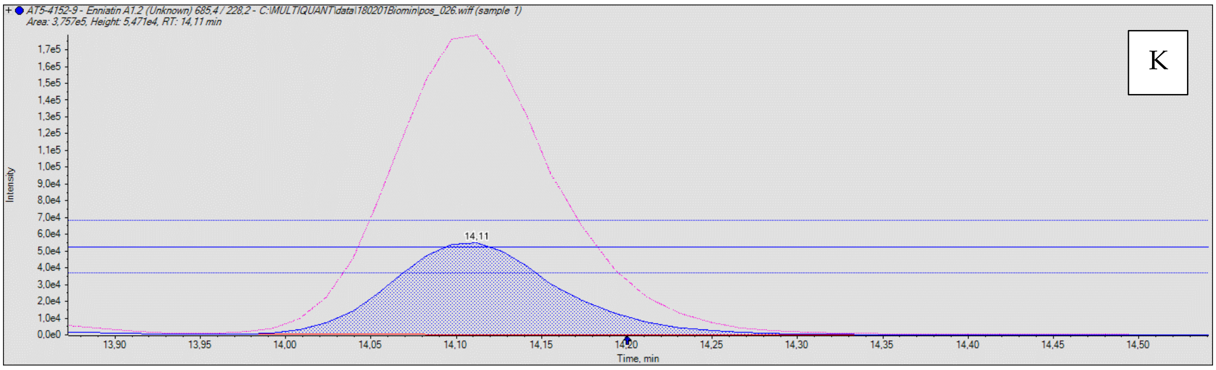

Figure S6. Overlay of the Extracted Ion Chromatograms of both quantifier (pink) as well as qualifier (blue; filled) of the 11 investigated compounds in sample AT5-4152-9; the horizontal lines denote the target height of the qualifier (± 30% tolerance) calculated from the height of the quantifier and the target ion ratio calculated from the standards.

Table S1. Concentrations of DON and emerging mycotoxins converted in µM and the corresponding different ratios.

**Metabolites**	**Concentrations at Selected Quartiles (µm)**			**Ratio DON/Emerging Mycotoxin**		
	P25	P50	P75	Ratio 1	Ratio 2	Ratio 3
DON	0.38	0.70	1.31			
BRV-F	0.06	0.10	0.16	21.7	7.1	2.4
Cyclo	0.45	0.75	1.42	2.9	0.9	0.3
TRPT	0.74	1.22	1.98	1.8	0.6	0.2
AFN	0.15	0.37	0.96	8.7	1.9	0.4
EMO	0.01	0.02	0.04	122.7	36.5	9.9
ENN-B1	0.02	0.06	0.13	87.4	12.2	2.8
ENN-B	0.01	0.05	0.13	95.3	13.9	2.9
BEA	0.005	0.01	0.02	258.3	70.0	21.9
ENN-A1	0.01	0.02	0.05	184.0	29.4	7.7
API	0.006	0.01	0.02	236.2	63.4	20.5

The concentrations corresponding to the quartiles P25, median, P75 of emerging mycotoxins in µg/kg in pig feed were converted in µM assuming their dilution in 1 L of gastrointestinal fluid. Ratio 1 was calculated using the P25 (1st quartile) concentration of emerging mycotoxin and P75 (3rd quartile) concentration of DON. Ratio 2 was calculated using the median (2nd quartile) concentration of DON and each emerging mycotoxin. Ratio 3 was calculated using the P75 concentration of emerging mycotoxin and P25 concentration of DON.
